# Antivirals against the Chikungunya Virus

**DOI:** 10.3390/v13071307

**Published:** 2021-07-05

**Authors:** Verena Battisti, Ernst Urban, Thierry Langer

**Affiliations:** Department of Pharmaceutical Sciences, Pharmaceutical Chemistry Division, University of Vienna, A-1090 Vienna, Austria; verena.battisti@univie.ac.at (V.B.); ernst.urban@univie.ac.at (E.U.)

**Keywords:** Chikungunya virus, alphavirus, antiviral therapy, direct-acting antivirals, host-directed antivirals, in silico screening, in vivo validation, antiviral drug development

## Abstract

Chikungunya virus (CHIKV) is a mosquito-transmitted alphavirus that has re-emerged in recent decades, causing large-scale epidemics in many parts of the world. CHIKV infection leads to a febrile disease known as chikungunya fever (CHIKF), which is characterised by severe joint pain and myalgia. As many patients develop a painful chronic stage and neither antiviral drugs nor vaccines are available, the development of a potent CHIKV inhibiting drug is crucial for CHIKF treatment. A comprehensive summary of current antiviral research and development of small-molecule inhibitor against CHIKV is presented in this review. We highlight different approaches used for the identification of such compounds and further discuss the identification and application of promising viral and host targets.

## 1. Introduction

Chikungunya virus (CHIKV) is a mosquito-borne alphavirus and belongs to the *Togaviridae* family. The virus was first isolated from a febrile patient in 1952/53 in the Makonde plateau (Tanzania) and has been named after the Makonde word for “that which bends you up”, describing the characteristic posture of patients suffering severe joint pains due to the CHIKV infection [1]. In the following years, only local and periodic outbreaks have been documented. However, in 2004 the CHIKV re-emerged at the coast of Kenya, spreading to La Reunion Island and surrounding island in the Ocean and South Asia [2]. A new CHIKV mutation with the A226V amino acid variant in the envelope glycoprotein E1 (CHIKV 06.21) was reported during that outbreak. This mutation, along with specific mutations in the E2 protein, allowed the virus to expand its vector potential from primarily *Aedes aegypti* to the more global *Aedes albopictus* and thus permitting the virus to spread in different temperature zones [3].

Consequently, in 2013 a CHIKV outbreak was reported on the Caribbean island St. Martin, following Brazil in 2014 and afterwards the rest of the American continent –causing more than 1.2 million cases of CHIKV infection in one year [4,5]. Furthermore, the first autochthonous outbreaks started to occur also in Europe (Italy and France) [6,7,8,9]. Due to globalisation, climate change, and lack of immunity in the worldwide population, this global spreading of the CHIKV is still ongoing-reaching new territories and higher numbers of infections [10,11,12].

Infection with the Chikungunya virus causes a high onset of fever for 3 to 10 days, followed by rash, myalgia, nausea, and severe joint pain [12]. Although this Chikungunya Fever (CHIKF) is rarely fatal, in approximately 50% of infected patients, the severe arthralgia and myalgia can last for months to years even after clearance of the viral infection [13]. However, the precise mechanism of these chronic CHIKF symptoms is still unclear. Furthermore, complications in patients with comorbidities and the elderly have been reported [14]. Up today there are no licensed drugs or vaccines available, and the alleviation of symptoms by, e.g., NSAIDs, is the only possible treatment for CHIKF patients [15].

## 2. CHIKV Replication Cycle

Like other alphaviruses, the entry of the CHIKV in cells involves the initial interaction of viral proteins with attachment factors and specific receptors from the host cell [16]. The CHIKV virion’s surface contains 80 trimeric spikes of E1 and E2 glycoproteins [17]. E2 facilitates the viral attachment to the host cell by interacting with surface host receptors followed by clathrin-mediated endocytosis (CME). The fusion of the viral membrane with the membrane of the host is triggered by a low pH environment leading to conformational changes of the viral envelop glycoprotein E1 [17]. Subsequently, the viral nucleocapsid is released into the cytoplasm, where it is disassembled to release the viral genome. The viral genome is then translated by the host cell translation machinery creating the non-structural-polyprotein P1234, which is cleaved into the precursor P123 and the viral non-structural-protein nsP4 [18]. P123 and nsP4 form the early replication complexes (RCs). They are responsible for synthesising the negative-strand RNA as a template to synthesise the desired positive-strand genomic RNA and sub-genomic RNA (26S RNA). Eventually, the formation of the P123 and nsP4 reaches a concentration threshold and the cleavage of the precursor P123 into fully processed nsPs is triggered. The 26S RNA, on the other hand, serves as the mRNA encoding the structural viral proteins C-pE2-6K-E1 [19,20,21].

Once formed, the capsid protein (C) is released by its autocleavage activity, while the remaining pE2-6K-E1 precursor is processed in the endoplasmic reticulum (ER) into pE2, 6K, and E1. While pE2 and E1 form heterodimer complexes, the cleaved capsid protein binds newly synthesised viral RNA, initiating to form the nucleocapsid core. This complex migrates towards the cell membrane through the Golgi secretory pathway, where pE2 is cleaved by the host enzyme furin or furin-like proteinases into the mature E2 and E3. E2 and E3 interact with the already formed nucleocapsid core and encapsidate the remaining viral RNA genome. Finally, the nucleocapsid core is recruited to the cell membrane where the particle buds from the cell where a new replication cycle begins [21,22,23].

## 3. Strategies for Identification of Antiviral Compounds

Various approaches have been utilised to identify antiviral compounds, such as cell-based high-throughput screening (HTS) and computational methods, including rational structure-based drug design on known crystal structures or homology models of viral and proviral host proteins. The most conventional method is the cell-based HTS with the virus-induced cytopathic effect (CPE) as readout. This method has the advantage of providing two simultaneous data points—the antiviral activity of the screened compounds and their cytotoxicity. Different compound libraries were used for the in silico and in vitro screening, ranging from libraries containing only FDA-approved drugs and libraries with compounds showing special chemical features to fragment-based libraries. The fast and economic computer-aided drug design has also been widely used for the identification of novel lead compounds by virtual screening. The identified hits were often further optimised by structure–activity relationship (SAR) assays. In addition, resistance selection in the presence of a compound was often performed in cell-based assays to identify the target protein of such compounds and give valuable insight into the viral pathways. 

Theoretically, all involved factors of the viral replication cycle could be potential targets for antiviral compounds. Like other viruses, the CHIKV uses a variety of interactions of viral proteins and host factors for its replication. The known and utilised proviral host factors are discussed below. Targeting such a host factor could provide broad-spectrum antiviral compounds as many viruses use the same replication strategies. On the other hand, unwanted side effects are more often seen in compounds with such an approach. Therefore, a combination of antiviral compounds with a different mechanism of action could provide a synergistic effect, leading to the reduction of antiviral drug concentration and thus may help decrease serious side effects. Moreover, such a combination could prevent the formation of drug resistance. 

## 4. Virus Targeting Inhibitors

A comprehensive overview of small molecules targeting viral proteins is given in Table 1. Furthermore, compounds with suggested viral target but without confirmation of the mode of action are summarized in Table 2. Studies without any in vivo or in vitro data were excluded. It is worth mentioning that the values given in Table 1 and Table 2 are not directly comparable to each other as they performed experiments, and setups differed between the discussed studies. Different virus strains, readouts (e.g., CPE and virus titer reduction) and cell lines were, for example, used and influence the assay results. 

### 4.1. Viral Entry and Membrane Fusion

**Figure 1:**
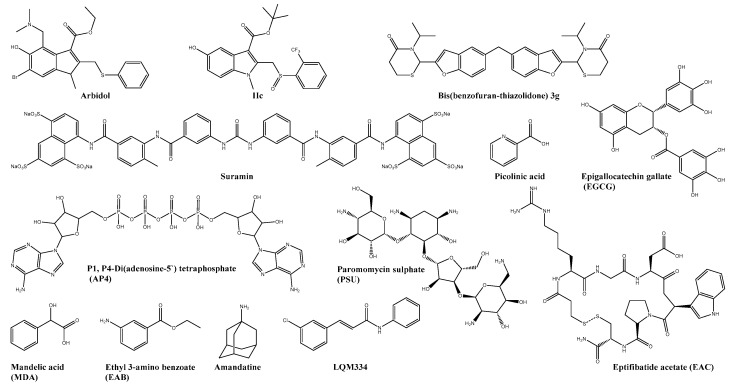
Chemical structure of virus-targeting compounds. Inhibitors of viral entry and membrane fusion: arbidol, IIc, suramin, epigallocatechin gallate, bis(benzofuran-thiazolidone) 3g, and LQM334. Inhibitor of the viral capsid protease: picolinic acid, mandelic acid, ethyl 3-aminobenzoate, P1, P4-di(adenosine-5′) tetraphosphate, eptifibatide acetate, and paromomycin sulphate. Inhibitor of 6K Protein: amantadine.

Many different factors and, therefore, potential targets are involved in the viral entry and fusion of the CHIKV, making it a widely used target for many antiviral compounds. The broad-spectrum antiviral drug arbidol (Figure 1), also known as umifenovir, and its metabolites, have shown to be early-stage inhibitors of CHIKV replication in different cell lines. The mode of action was confirmed by selecting an arbidol-resistant variant carrying an arginine (G407R) mutation localised in the viral E2 glycoprotein–a type I transmembrane protein involved in the virus binding to the host membrane [24]. Indole-based arbidol analogues with sulfoxides and tert-butyl esters have demonstrated an increased potency and selectivity index. Although docking studies with the most promising analogue IIc (Figure 1) identified two potential binding sites in E2, a post entry inhibition of CHIKV was observed in a time of addition assay. Furthermore, IIc was ~6-fold less potent than arbidol in an entry assay with CHIKV pseudoparticles—suggesting that IIc may have a different mode of action [53,64].

Suramin (Figure 1), a symmetrical sulfonated naphthylurea compound and an FDA approved drug against trypanosomiasis, has also demonstrated to inhibit the early-stage of the CHIKV replication cycle in different independent studies–using not only time of addition assays but also in silico methods [25,27,65]. A more detailed investigation of the mechanism of action was performed by Albulescu et al., where suramin was found to interact directly with the viral particles of CHIKV by inhibiting the viral attachment to the host membrane and interfering with the fusion step by suppressing the conformational changes of viral envelope glycoproteins [66]. Moreover, suramin showed a synergistic effect with the green tea catechin epigallocatechin gallate (EGCG, Figure 1)—a known in vitro early-stage inhibitor of CHIKV infection [67,68]. Although an in vivo study with CHIKV-infected C57BL/6 mice demonstrated amelioration of CHIKV-induced foot swelling, inflammation, and cartilage damage, clinical trials have shown severe side effects during long term treatment of suramin [26,69,70]. Additionally, the suramin-based analogues with the chemical structures of bis(benzofuran-thiazolidinone)s (Figure 1) and bis(benzofuran-thiazinanone)s demonstrated a 29–42 fold more potent anti-CHIKV activity than suramin, but their high toxicity remains an unsolved issue [54]. 

An in silico screening experiment based on a molecular docking approach utilising a variety of biological targets of CHIKV and the following in vitro evaluation by an MTT assay of the obtained hits identified LQM334 (Figure 1) as a promising inhibitor of CHIKV. The mode of action of the newly found lead compound remains unclear, but a molecular docking study indicates a possible interaction between LQM334 and the E2 domain A from the mature E3-E2-E1 glycoprotein complex [55]. In addition, Agarwal et al. performed an in silico docking study using the structure of the envelope glycoprotein of CHIKV to identify promising new lead compounds, but the in vitro confirmation of their antiviral activity has still to be shown [71]. 

Micafungin, an FDA approved drug to treat candidiasis, showed a broad spectrum of inhibitory effects against different alphaviruses—including SINV, SFV and CHIKV. Although a molecular docking study indicated a possible interaction with the CHIKV envelope glycoprotein, a time of addition assay also pointed to a late-stage inhibition of the CHIKV infection, indicating an inhibitory effect against viral replication, extracellular and cell-to-cell transmission of CHIKV [56].

### 4.2. Capsid Protease

The pyridine containing compound Picolinic acid (PCA, Figure 1) is a known antiviral against various viruses, including the alphavirus SINV. Furthermore, PCA was demonstrated to bind the hydrophobic region of CHIKV capsid protein, interfering with the interaction of the cytoplasmic domain of E2 glycoprotein (cdE2) and the capsid, which is needed to facilitate the budding of the virus from the plasma membrane of the host cells [28,72]. A significant reduction in vRNA levels and infectious virus was observed when treated with PCA [28]. The same research group used these findings in combination with other studies about proposed capsid protease inhibitors such as dioxane and piperazine to perform an in silico screening for potential new lead compounds targeting the capsid protease [28,73,74,75,76]. Their most promising hits (S)-(+)-mandelic acid (MDA, Figure 1) and ethyl 3-aminobenzoate (EAB, Figure 1) showed better binding tendencies than dioxane and PCA in silico, but the in vitro evaluation of their antiviral activity is still pending [76]. More recently, a structure-assisted drug-repositioning study based on in silico screening and ranking of the hits by their docking score identified three compounds targeting the auto-proteolytic activity of the capsid protease: P1, P4-Di(adenosine-5′) tetraphosphate (AP4, Figure 1), eptifibatide acetate (EAC, Figure 1) and paromomycin sulphate (PSU, Figure 1) [58]. 

### 4.3. 6K Protein

The potential of the 6K protein as a possible target for antiviral drug development demonstrates the potent antiviral drug amantadine. This FDA approved anti-influenza drug targets the ion channel-forming M2 viroporin of the influenza virus [77]. Furthermore, electrophysiology experiments indicated that amantadine hinders the ion channel activity of CHIKV 6K and alters the morphology of CHIKV virus-like particles. The anti-CHIKV potential of amantadine was shown in infected Vero cells [29].

### 4.4. Non-Structural Proteins

#### 4.4.1. Non-Structural Protein 1 (nsP1)

**Figure 2:**
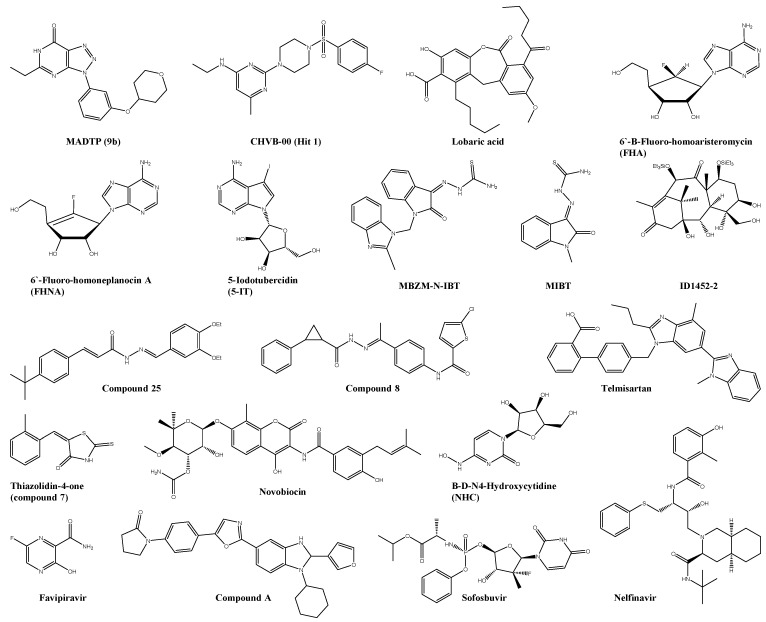
Chemical structure of virus-targeting compounds. Inhibitors of nsP1: MADTP, CHVB, lobaric acid, 6′-β-fluoro-homoaristeromycin, 6′-fluoro-homoneplanocin A, and 5-iodotubercidin. Inhibitor of nsP2: compound 25, compound 8, MBZM-N-IBT, MIBT, thiazolidin-4-one, ID1452-2, telmisartan, novobiocin, and nelfinavir. Inhibitor of nsP4: favipiravir, β-D-N4-Hydroxycytidine, sofosbuvir, and compound-A.

The first class of small molecules reporting the nsP1 of CHIKV as the potential target is the MADTP series (Figure 2), with a triazolopyrimidinone scaffold and MADTP-314 as the initial lead compound [30,31,32,33]. Overall, three consecutive structure–activity-relationship studies were performed-aggregating detailed information about the influence of various structural changes and demonstrating potent inhibitory effects on various CHIKV strains and VEEV nsP1 in an enzymatic assay [30,32,33]. The selection of a MADTP-resistant CHIKV strain in cell culture and the following reverse genetics identified the single-amino-acid substitution P34S in the GTase functional domain of nsP1 as responsible for the MADTP-resistance [31]. Recently, 2-(4-(Phenylsulfonyl)piperazine-1-yl)pyrimidine analogues, i.e., the CHVB series (Figure 2), were identified as potent and selective anti-CHIKV compounds and analysed based on their structure–activity relationship [34]. In addition, CHVB compounds showed potent inhibitory effects of the MTase and GTase activities of nsP1 of Semiliki Forest virus (SFV) and VEEV [35]. Interestingly, a CHVB-resistant virus demonstrated cross-resistant to the MADTP series, suggesting that both compound families utilise a similar mode of action. However, the CHVB series required the presence of at least two mutations in nsP1, namely, S454G and W456R, indicating that the barrier of resistance is higher for the CHVB series and the occurrence of resistance in clinical settings is less likely [35]. 

A fluorescence polarisation-based assay measuring the GTP binding site was used to perform an HTS and led to the identification of a series of hits [36]. The cherrypicked hits were subsequently not only evaluated with an orthogonal assay to measured their ability to interfere with the guanylation step of the capping reaction of nsP1 but also tested for their antiviral activity [36]. These findings led to the identification of the naturally-derived compound, lobaric acid (Figure 2), as a potent CHIKV nsP1 inhibitor [36]. 

More recently, two carbocyclic adenosine analogues, namely, 6′-β-fluoro-homoaristeromycin (FHA, Figure 2) and 6′-fluoro-homoneplanocin A (FHNA, Figure 2), have been identified as inhibitors of the MTase activity of nsP1 by screening a library designed to inhibit the host target SAH hydrolase (see 5.5.1.1. Hydrolases) [37,38]. Resistance selection unveiled two mutations G230R and K299E in nsP1 to develop resistance against both compounds [38]. Additionally, 5-iodotubercidin (5-IT, Figure 2) showed also an inhibitor of the MTase activity of nsP1 in a capillary electrophoresis-based assay [39]. This derivate of tubercidine and a known adenosine kinase inhibitor demonstrated potent anti-CHIKV activity in a plaque-reduction assay [39].

#### 4.4.2. Non-Structural Protein 2 (nsP2)

The nsP2 has become a significant target of interest in the development of anti-CHIKV drugs primarily because of its essential role in the CHIKV replication but also because of its relatively early publication of its 3D structure (PDB: 3RTK) [78]. This led to a significant number of publications focussing on the in silico approach to identify potential new nsP2 inhibitors [79,80,81,82,83,84,85,86,87]. However, for all of those hits predicted in these studies, no in vitro evaluation was made. This vital evaluation step was made by Bassetto et al. by the combination of virtual screening with an optimised homology model of nsP2 and the following evaluation by virus-cell-based CPE reduction assay [40]. This approach led to discovering the first lead compound, compound 25 (Figure 2), with potential nsP2 protease inhibition activity. Two following structure–activity-relationship studies with altogether 100 analogues were additionally performed by this research group to determine the compound series’s critical components and investigate the impact of some chemical-structure changes on antiviral activity and water solubility [41,42]. Interestingly, molecular docking on nsP2 showed no significant differences in binding mode between the most active compound (compound 25 with EC_50_ = 3.2 μM) and some of its poorly active analogues. In their virtual screening study based on pharmacophoric features of the initial hit of Bassetto et al., Das et al. describe a set of 12 compounds with anti-CHIKV activity with the most potent inhibitor compound 8 (EC_50_ = 1.5 μM, Figure 2) [40,43]. A cell-free protease assay was performed to verify their effects on nsP2, in which the majority of the compounds demonstrated inhibitory effects against nsP2 [43]. Surprisingly, the initial hit of Bassetto et al. did not show any inhibition [40,43]. However, three analogues showed significant inhibitory effects, pointing to two different sites of actions in the compound series [41,43]. This example highlights the significance of biological evaluations of in silico predictions as they may sometimes not correlate with the in vitro results. On the other hand, molecular docking and molecular dynamic simulations may be an economical and fast starting point to investigate a possible mode of action of potential new antiviral drugs. Mishra et al. used such a computational approach to examine the target of their antiviral compounds MBZM-N-IBT and MIBT (Figure 2) [59]. Although their docking result points to an nsP2 inhibition, a time-of-addition assay revealed an early stage and a late-stage inhibition of the CHIKV–indicating multiple modes of actions. Another study described five arylalkylidene derivates of 1,3-thiazolidin-4-one (Figure 2) with low micromolar antiviral concentrations and interactions with the nsP2 protease domain in MD-simulations [60].

Small peptidomimetics were discovered using quantum mechanics-based ligand descriptors and biologically evaluated in virus-cell-based assay [61]. Docking of the most potent analogues, peptidomimetic 3a/4b, with the crystal structure of nsP2 exposed the advantage of lower molecular weight in this compound series, likely due to their more accessibility to the target pocket [61]. Other peptidomimetics, PEP-I and PEP-II, were identified by screening based on pharmacophoric features derived from nsP2-nsP3/4 (protein-peptide) interaction and evaluated by their anti-CHIKV activity using plaque reduction assay [62,88]. Their inhibitory effect on chikungunya nsP2 protease and the proteolytic activity of nsP2 was investigated by a FRET-based protease assay [62]. Both compounds inhibited their target protein in the micromolar range [62]. 

The natural compound, ID1452-2 (Figure 2), was discovered by high-throughput screening to identify small molecules targeting the chikungunya nsP2 protease [44]. ID1452-2 showed moderate anti-CHIKIV activity (IC_50_ = 31 μM) and inhibited nsP2 effects in dose-dependent manner [44]. More recently, a target-based drug screening with 30,000 FDA approved molecules and SPR experiments identified telmisartan (Figure 2), an antihypertensive drug, and the antibiotic novobiocin (Figure 2) as a strong inhibitor of the chikungunya nsP2 protease [45]. Both drugs inhibited the nsP2 protease activity in low micromolar concentrations [45]. This strategy of repurposing of FDA approved drugs were also used by Bhakat et al. to accelerate the identification and development of future anti-CHIKV drugs [63]. MM/GBSA-based binding free energy results and molecular docking on nsP2 determined nelfinavir (Figure 2), an HIV/HCV inhibitor, as a potential new anti-CHIKV drug [63]. This compound showed a moderate antiviral effect in the CPE-reduction assay [63]. 

#### 4.4.3. Non-Structural Protein 3 (nsP3)

No small molecules targeting CHIKV nsP3 are reported up to date. Recently, a fragment library and x-ray crystallography screening led to the discovery of 40 fragments binding to the distal ribose binding site of ADP-ribose in the nsP3 macrodomain crystal structure (PDB-code: 6VUQ) [46]. As most of the fragments share a similar pyrimidine-based scaffold, it could be an interesting starting point for further development of a CHIKV nsP3 inhibitor [46]. A similar approach to identify small molecules with anti-nsP3 activity was conducted by Nguyen et al. [89]. With virtual screening and molecular docking, various hits were detected, but the ability of the compounds to inhibit the CHIKV in vitro has not yet been published [89].

#### 4.4.4. Non-Structural Protein 4 (nsP4)

The nsP4 protease functions as an RNA-dependent polymerase (RdRp) and is the most highly conserved protein in the alphavirus family [90]. Consequently, many compounds targeting this protease have been reported to inhibit the CHIKV and other alphavirus replication cycles. Favipiravir (T-705, Figure 2) and its defluorinated analogue T-1105, for example, were reported to inhibit in vitro replication of different CHIKV strains as well as other (arthritogenic) alphaviruses [47]. All favipiravir resistant CHIKV strains carried a unique K291R mutation in a highly conserved F1 motif of the RNA-dependent RNA polymerase of +ssRNA viruses in nsP4 [47]. This highly conserved lysine is crucial for the anti-CHIKV activity and responsible for the broad-spectrum antiviral activity of T-705 [91]. In addition, a CHIKV-mouse model of lethal infection in AG129 mice, favipiravir treatment (300 mg/kg/day for seven days) prevented the development of severe neurological disease and increased the survival rate [47]. Further, favipiravir treatment (300 mg/kg/day for four days) in C57BL/6J mice reduced the viral replication in the joints when administered in the acute phase and prevented systemic viral spread [48]. Recent findings highlight the influence of different cell lines on the antiviral outcome in biological assays of T-705 and the impact of various CHIKV strains on the disease severity in mouse strains, and the efficacy of favipiravir treatment [92,93]. 

Another nucleoside analogue, β-D-N^4^-hydroxycytidine (NHC, Figure 2), showed more potent anti-CHIKV effects in Vero Cells than the control nucleoside analogues favipiravir and ribavirin [49]. The alphavirus VEEV needs for the development of even low-level resistance against NHC multiple cooperative mutations within the RdRp of nsP4 [50]. Additionally, NHC has shown in a time-of-addition assay to have no effect on viral entry but in the early stage of the CHIKV replication circle [49]. Both findings indicate the RdRp domain of nsP4 as the potential target of NHC, but the precise mode of action remains unclear. 

Sofosbuvir (Figure 2), the FDA approved drug against hepatitis C virus, and uridine analogue also showed interesting activity against CHIKV in different cell lines [51]. Additionally, treatment with sofosbuvir in the CHIKV-mouse model of adult Swiss mice (20 mg/kg/day) protected against CHIKV-induced disease and increased the survival rate of neonate mice (40 and 80 mg/kg/day) [51].

Although the nsP4-targeting compounds have shown to be primarily nucleoside analogues, a high throughput screening of chemical compound libraries identified compound-A (Figure 2) as a CHIKV infection inhibitor [52]. This benzimidazole-related compound inhibited several different CHIKV strains and SINV strains in Vero cells [52]. A key mutation for development compound-A resistance was identified in a mechanism of action study–the M2295I residue located in the functional domain of RdRp of nsP4 [52]. Kumar et al. conducted an in silico study on a CHIKV nsP4 homology model to identify potential new hits. However, the biological evaluation of their collected data has yet to be done [94].

## 5. Targeting Host Factors

As discussed above, a variety of proviral host factors have been shown to influence the viral replication cycle. Therefore, many small molecules modulating such factors have been reported to possess antiviral activity. A comprehensive overview of anti-CHIKV compounds with proviral host targets is given in Table 3. The same criteria for inclusion and issues with data comparison as in Table 1 and Table 2 (see Section 4, Virus Targeting Inhibitors) are also applied here. 

### 5.1. Viral Entry and Membrane Fusion

Alphaviruses use a receptor-mediated endocytotic entry and pH-dependent fusion to release their viral RNA genome into the host cell cytoplasm. Recent findings in biochemistry and structural identification of involved proteins have given a valuable insight into this process, where many different host factors could be used as potential antiviral targets [16].

**Figure 3:**
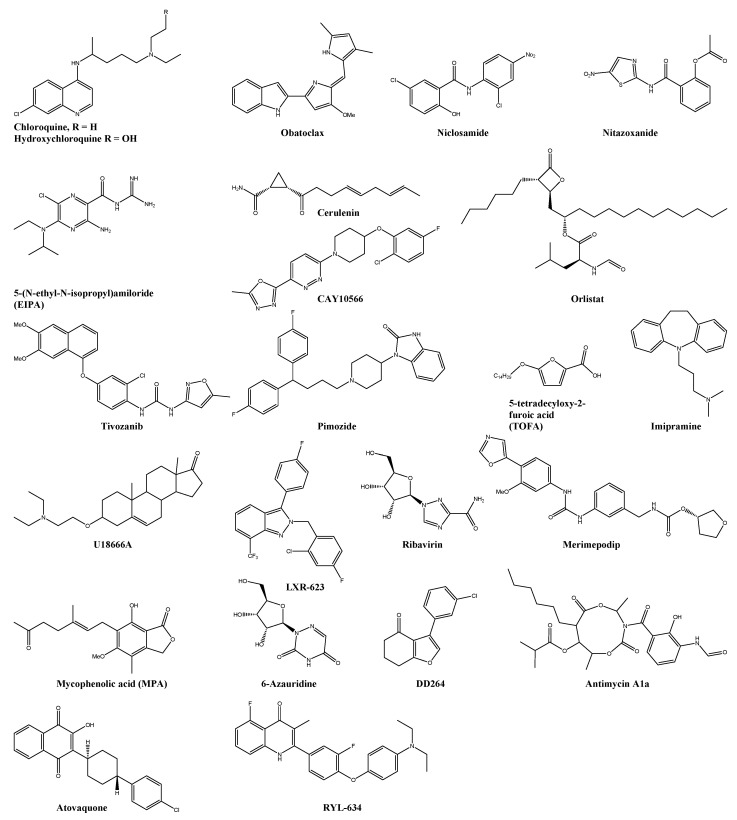
Chemical structure of selected host-targeting compounds. Inhibitor of the viral entry and membrane fusion: chloroquine, hydroxychloroquine, obatoclax, niclosamide, nitazoxanide, and 5-(N-ethyl-N-isopropyl)amiloride. Inhibitor of the lipid pathway: orlistat, cerulenin, CAY10566, tivozanib, pimozide, 5-tetradecyloxy-2-furoic acid, imipramine, U18999A, and LXR-623. Inhibitor of the pyrimidine and purine synthesis: ribavirin, merimepodip, mycophenolic acid, 6-azauridine, DD264, antimycin A1a, RYL-634, and atovaquone.

One of such antiviral compounds is chloroquine (Figure 3), a 9-aminoquinoline known since 1934 as an antimalarial drug [141]. It has been used in clinical trials against CHIKV even long before its anti-CHIKV effect was reported in vitro assays. The reasoning behind this unique approach lies in the lack of other treatment options and promising research results published before. Chloroquine and other NSAIDs such as mefenamic acid (see Section 5.3, Pyrimidine and Purine Synthesis Inhibitors) have been reported to inhibit the multiplication of various viruses (e.g., Sindbis, influenza A2, herpes simplex, etc.) in chick and mouse embryo cells [142,143]. Additionally, it has been shown to influence the pH-dependent fusion of the Sindbis virus and Semiliki Forest virus with the endosomal membrane by raising the endosomal pH in BHK-21 cells [144,145]. Other weak bases such as NH_4_Cl, amantadine, methylamine and tributylamine showed similar lysosomotropic effects [144,145]. Moreover, chloroquine has been reported to lessen the joint inflammation of patient with rheumatoid arthritis in several trials in the 1950s [146]. The first clinical trial 1984 with chloroquine phosphate on 10 CHIKF patients was conducted after empirical observations that one of the patients joint pains improved while taking antimalarial drugs prophylactically [147]. Treatment with chloroquine led to alleviating patients’ symptoms and opened the door for further clinical trials [147]. However, a randomised, double-blind, placebo-controlled, prospective trial (CuraChik trail) with 54 adult patients diagnosed with CHIKV showed no significant difference between the placebo and the chloroquine groups in terms of fever clearance time or viremia clearance time [148,149]. Moreover, patients treated with chloroquine were more likely to complain about persistent arthralgia (*p* < 0.01) and suffered moderate adverse effects of the treatment [148,149]. Another clinical trial showed no advantage of chloroquine treatment over the NSAID meloxicam in patients with early musculoskeletal pain and arthritis following acute chikungunya virus infection [150]. While the in vivo performance of chloroquine in humans and non-human primate models is limited, the in vitro effects of chloroquine are remarkable better [95,151,152,153]. These contradictory findings may be due to the immunomodulatory effects of chloroquine in vivo: it inhibits, i.e., IFN-I responses, which may influence the immune response to the viral replication negatively and may have been missed in the used Vero-E6 cells, which do not produce IFN-I [152].

Several clinical trials were conducted to investigate hydroxychloroquine (HCQ, Figure 3) on Chikungunya virus infection [154]. HCG seems to have no beneficial impact in the early stage of CHIKV infection or reduction of joint pain when administered alone or in combination with aceclofenac [155]. However, the combination of HCQ with methotrexate improved the disease activity and reduced disability and pain in patients [156,157]. In contrast, HCQ treatment in the RHUMATOCHIK study had to be interrupted in 4 out of 39 patients because of adverse effects such as nausea, rash, stomatitis, and headache [158]. A 50% reduction of synovitis and 19.2% complete remission was reported in the remaining sample [158].

The pH-dependent endocytosis and the high sensitivity of CHIKV to the antiviral activity of type I and type II interferons was also shown by Sourisseau et al. [159]. Their study demonstrated the anti-CHIKV effect of chloroquine and the vacuolar proton ATPase inhibitor bafilomycin-A. Furthermore, the high sensitivity of CHIKV to the antiviral effect of IFNs (see Section 5.7, Immunomodulatory) has been reported [159]. Concamycin A, another vacuolar proton ATPase inhibitor, and the Bcl-2 inhibitor obatoclax (Figure 3) inhibited the viral fusion of Semiliki forest virus and, in the case of obatoclax, also of CHIKV due to their lysosomotropic characteristics [96,160].

A broad range of antiviral effect on pH-dependent viruses demonstrated the anithelmic drugs niclosamide and nitazoxanide (Figure 3) [161]. Radiometric imaging allowed the precise measurement of endosomal pH and revealed their neutralising effect of acidic endosomes [161]. An additional structure–activity relationship study exposed the importance of the hydroxy and chloride group in position R1 and R4 for the antiviral effect of this compound series [161]. An HTS for CHIKV fusion inhibitors with FDA approved drugs found not only niclosamide and nitazoxanide as potential anti-CHIKV candidates but also suramin (see Section 4.1., Viral Membrane Fusion and Entry) [97]. All of them showed an additional inhibitory effect on the cell-to-cell transmission of CHIKV. Additionally, the antiviral effect of the hits was evaluated, measuring the CHIKV-induced CPE in BHK-21 cells, and no toxicity in the used micromolar range was observed in zebrafish embryos [97].

Pre-infection treatment with the macropinocytosis inhibitor 5-(N-ethyl-N-isopropyl)amiloride (EIPA, Figure 3) results in a dose-dependent inhibition of CHIKV infection [98]. Amiloride and an amiloride analogue HOE-694 have been reported to block the activity of Na(+)/H(+) exchanger, lowering the submembranous pH and consequently preventing the necessary macropinocytosis [162]. Macropinocytosis has been identified as a significant pathway of CHIKV into muscle cells and is, therefore, an attractive target for new anti-CHIKV compounds [98].

### 5.2. Lipid Pathway Inhibitors

Alphavirus fusion is dependent on the cholesterol and sphingolipid in the host cell membrane [16]. Consequently, inhibition of the fatty acid synthase by the anti-obesity drug orlistat, the antibiotic FASN inhibitor cerulenin, and the SCD1 inhibitor CAY10566 resulted in decreased CHIKV and MAYV genome replication (see Figure 3) [99,100]. Both enzymes play a central role in the de novo synthesis of long-chain fatty acids and are crucial in the replication of various viruses [100]. Furthermore, orlistat was reported as a potential broad-spectrum agent against mosquito-transmitted viruses such as DENV, JEV, ZIKV and CHIKV [101].

Additionally, the fatty acid synthase was identified by Bakhache et al. as an important proviral host factor [100]. Further, a genome-wide CHIKV/HEK-293 loss-of-function siRNA screen led to identifying 156 proviral and 41 host targets influencing the CHIKV replication cycle [99]. The validated host proviral factors were used to screen a drug repurposing database [99]. This approach led to the identification of 20 compounds interacting with six unique host targets, including enzymes of fatty acid synthesis (fatty acid synthase, ATP citrate lyase, and acetyl CoA carboxylase), calmodulin signaling, the vacuolar-type H^+^ ATPase (vATPase), CLK1, fms-related tyrosine kinase 4 (FLT4 or VEGFR3), and K (lysine) acetyltransferase 5 (KAT5 or TIP60). All 20 compounds showed strong antiviral activity when tested in HEK293-T cells, but, as expected, some of them exhibited a narrow therapeutic index. The in vitro outcome was further validated in a CHIKV-C57BL/6 mouse model, in which tivozanib (targeting FLT4), pimozide (calmodulin inhibitor) and 5-tetradecyloxy-2-furoic acid (TOFA, fatty acid synthesis inhibitor) reduced the viral replication in the footbed significantly (see Figure 3). The combination of TOFA and pimozide had a synergistic effect in reducing the viral replication and joint swelling [99].

The host membrane cholesterol is a key component in the unmasking of the fusion peptide in class II envelope glycoproteins [102]. Compounds interfering with the cholesterol transport such as the tricyclic antidepressant imipramine (Figure 3) and the class II cationic amphiphilic compound U18666A (Figure 3) have shown to affect the fusion and replication step from not only the CHIKV but also several Flaviviridae such as ZIKV, West Nile virus, and DENV [102].

The ubiquitously expressed liver X receptors (LXRs) are essential in the regulation of cholesterol homeostasis and a potential proviral host target for antiviral compounds [103]. The selective, synthetic agonist of LXRβ LXR-623 (Figure 3) inhibited the CHIKV replication in human foreskin fibroblasts in a dose-dependent manner. This effect was partially reversed when the cells were incubated with cholesterol [103].

### 5.3. Pyrimidine and Purine Synthesis Inhibitors

The inosine monophosphate dehydrogenase (IMPDH) is a key enzyme in the de novo guanine nucleotide biosynthesis by converting inosine-5′-monophosphate to xanthine 5′-monophosphate and is, therefore, an interesting target for antibacterial, anticancer and antiviral drugs [163]. Ribavirin (Figure 3), an FDA-approved drug against respiratory syncytial virus infection in infants and chronic hepatitis C infection, is a guanosine analogue with multiple postulated biomechanisms [164,165]. The inhibition of IMPDH leading to depletion of GTP pools as well as the inhibition of the RNA-dependent RNA polymerase (RdRp) is considered the major causes of the broad-spectrum antiviral activity of ribavirin [165]. The scientific research about the precise mode of action is still ongoing, but a time-of-addition study unveiled that ribavirin is primarily active at the early stage of the CHIKV replication cycle [59]. Ribavirin showed antiviral activity against CHIKV in vitro and in vivo studies and exhibited a synergistic effect with the tetracycline doxycycline, IFN α2a, and the NSAID mefenamic acid (MEFE) [57,104,166,167]. Moreover, a 7-day treatment with 200 mg ribavirin twice daily significantly improved joint pains in human patients [168].

Another potent and selective IMPDH inhibitor, merimepodib (Figure 3), inhibited the CHIKV and the ZIKV in a dose-dependent manner [105]. On the other hand, the immunosuppressive agent and non-competitive inhibitor of IMPDH, mycophenolic acid (MPA, Figure 3), has been reassessed regarding its anti-CHIKV inhibitory effect [169]. Although it has demonstrated high antiviral effects in a virus-induced cytopathic effect assay, 19 synthesised analogues of MPA did not show any CHIKV inhibition [169,170]. The following retest of MPA revealed the unexpected characteristic of MPA: after reduction of GTP and downregulation of CHIKV replication, the antiviral effect of MPA diminishes, and the virus regains its full replication potential [169].

6-Azauridine (Figure 3), on the other hand, inhibits the orotidylic acid decarboxylase (OMP)–resulting in depletion of UTP pools in cells [171]. It is used in the treatment of psoriasis and showed broad-spectrum antiviral activity [171,172,173]. Additionally, 6-azauridine has been reported to reduce the viral titer of CHIKV and SFV [104].

Another proviral host target is the dihydroorotate dehydrogenase (DHODH)–the fourth enzyme in the pyrimidine biosynthetic pathway bound to the inner mitochondria membrane [107]. DHODH is the suggested target of the DD264 series (Figure 3)—a small compound series found through an HTS with broad-spectrum antiviral activity (DNA and RNA viruses, including CHIKV) [106,107]. Interestingly, the antiviral effect of DD264 was suppressed when added exogenous uridine but not with added guanosine, which supports the theory that the pyrimidine level is essential for the CHIKV replication [106]. Antimycin A1a (Figure 3) also inhibits the cellular mitochondrial electron transport, suppressing the de novo pyrimidine synthesis and resulting in a broad spectrum of antiviral activity [174].

Phenotypic screening of around 200 biaryl-substituted quinolones and the following in vitro validation and SAR study led to the identification of the broad-spectrum antiviral compound RYL-634 (Figure 3) [108]. Via activity-based protein profiling (ABPP), 78 potential human proteins were identified as possible targets of RYL-634. Further in silico investigations and enzymatic activity assays validated the DHODH as the target enzyme [108]. Although it has been already shown that the antimalaria drug atovaquone (Figure 3) has an inhibitory effect on DHODH, its biomechanism regarding the antiviral effect on CHIKV remains unclear [109,175]. A mechanism of action study showed an early stage inhibition of ZIKV infection and a possible inhibitory effect on the pyrimidine biosynthesis pathway [109].

### 5.4. Protein Synthesis Inhibitors

**Figure 4:**
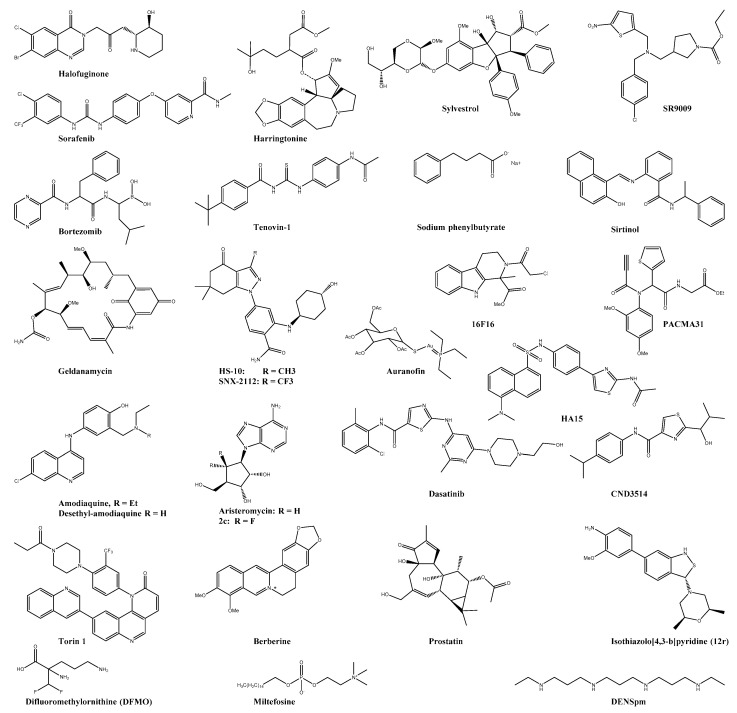
Chemical structure of selected host-targeting compounds. Inhibitor of the protein synthesis: halofuginone, harringtonine, sorafenib, sylvestrol, bortezomib, and SR9009. Inhibitor of cellular proteins: tenovin-1, sodium phenylbutyrate, sirtinol, geldanamycin, HS-10, SNX-2112, HA15, 16F16, PACMA31, and auranofin. Inhibitor of cellular enzymes: amodiaquine, desethyl-amodiaquine, aristeromycin, dasatinib, Torin 1, CND3514, berberine, miltefosine, prostatin, isothiazolo[4,3-b]pyridine, difluoromethylornithine, and DENSpm.

Halofuginone (Figure 4) is an antagonist of the host prolyl-tRNA synthetase enzyme (EPRS), causing the accumulation of uncharged tRNA^pro^ and forcing the cell to suppress the translation even when proline levels are sufficient [176]. This orally available synthetic derivative of the plant compound febrifugine has been reported to inhibit the viral progeny production of CHIKV, ONNV, ZIKV and DENV [110]. Harringtonine (Figure 4), another naturally derived compound, has been identified to inhibit CHIKV RNA production and viral protein expression in a dose-dependent manner [111]. This cephalotaxine alkaloid is known to inhibit eukaryotic translation [177].

The anticancer drug sorafenib tosylate (Figure 4) inhibited CHIKV replication at 8 and 16 h post-infection through dephosphorylation of several key enzymes for the viral translation-including the cap-binding protein eIF4E (eukaryotic translation factor 4E) and p70S6K [99,112]. As the research from McKendrick et al. suggest that the phosphorylation of eIF4E is not essential for the global cellular protein synthesis, this enzyme could be an attractive target for further antiviral drug development [112,178]. Moreover, sylvestrol (Figure 4), a specific inhibitor of the RNA helicase eIF4A (eukaryotic translation factor 4A), inhibited the CHIKV replication cycle at an early stage [179]. The DEAD-box helicase eIF4A unwinds the RNA secondary structure in the 5′-untranslated regions (5′-UTRs) of mRNA and allows translation. Silvestrol holds the eIF4A helicase to its mRNA substrate and inhibits thereby the following translation [179]. More recently, Blum et al. reported the influence of sylvestrol on the inflammatory status of immune cells [180].

The ubiquitin-proteasome system (UPS) is central for ensuring protein quality control and maintaining a critical level of important regulatory proteins [181]. As many viruses have evolved to manipulate this cellular machinery in their favour, it is not surprising that the FDA-approved proteasome inhibitor bortezomib (Figure 4) was reported to inhibit different CHIKV strains in various cell lines [113,181]. Investigation of the CHIKV protein level by Western plot analysis revealed a 50% to 80% reduction of E2, E1 and capsid protein [113]. The synthetic agonist of the nuclear receptors Rev-erb α/β SR9009 (Figure 4) showed inhibitory effects against the CHIKV and O′nyong′nyong virus [114]. Although the precise mechanism of action is still unclear, a subgenomic RNA translation inhibition was observed [114].

### 5.5. Cellular Protein Inhibitors

Sirtuins (SIRTs) are an evolutionarily conserved family of seven lysine deacetylases (KDACs) and are present in nuclear and cytoplasmic compartments [115]. Their precise functions in human cells are not fully elucidated, and their impact on viral replications varies not only on the viral pathogen but also on the subgroup of SIRTs itself [115]. Different sirtuin inhibitors, such as tenovin-1 (Figure 4), sodium phenylbutyrate (a pan-KDAC inhibitor, Figure 4), and sirtinol (a specific SIRT1 and SIRT2 inhibitor, Figure 4) have been reported to inhibit a set of flaviviruses, bunyaviruses, and alphaviruses–including the CHIKV. Interestingly, the inhibition of only SIRT1/2 was not enough to block the viral infection [115].

Like cellular proteins, viral proteins need chaperones for proper folding and assembling for precise and stable function. Three different chaperon families, namely the heat shock protein 90 (HSP90), the protein disulfide isomerase (PDI), and the ER chaperon GRP78, were reported as proviral host factors in the case of CHIKV infection [116,117,118]. HSP90 has a critical role in the proper folding, maturation, localisation, and turn-over of cellular and viral proteins. Its essential function for RNA and DNA viruses makes it a desirable host target for broad-spectrum antivirals [182]. Interestingly, the known HSP90 inhibitor geldanamycin (Figure 4) showed antiviral effects in CHIKV infected HEK-293T cells. Further, the antiviral effects of two specific HSP90 inhibitors, HS-10 and SNX-2112 (Figure 4), ruled out any off-target effects of geldanamycin during the CHIKV replication cycle. They even prevented joint swelling and inflammation in a CHIKV-SVA129 mouse model [116]. Further, geldanamycin treatment led to reducing the nsP2 concentration and the for viral replication essential interaction between HSP90 and nsP2 [183]. Another chaperon inhibitor, namely the compound HA15 (Figure 4), was recently found to inhibit various viruses—including VEEV, EEEV, SINV, and CHIKV. The ER chaperon GRP78 showed interactions with the viral E2 glycoprotein and was confirmed as a possible proviral host target [117].

Alphaviruses require specific disulfide bonding in their envelope proteins E1 and E2 for proper folding and assembling and, therefore, likely depend on the host protein disulfide isomerase (PDI) [118]. Consistent with these findings, the in vitro assays with the PDI inhibitor 16F16 (Figure 4) led to a significant reduction of cell-cell fusion events. Although 16F16 and PACMA31 (another PDI inhibitor, Figure 4) showed interesting antiviral effects, their toxicity profile was remarkably poor. However, the FDA approved TRX-inhibitor auranofin (Figure 4) had a much better therapeutic index of 104.5 at 12 h post-infection and even reduced food swelling in a CHIKV mouse model [118].

#### 5.5.1. Cellular Enzyme Inhibitors

##### Hydrolases

So far, three different hydrolase families have been reported as proviral host targets for the inhibition of the CHIKV infection, namely furin, cathepsin B, and the SAH-hydrolase [120,153,184]. The membranous furin hydrolase is vital for the cleavage of the alphavirus envelope glycoproteins E1 and E2 precursor P62 [153,185]. As this process is a crucial moment in replicating the CHIKV and many other alphaviruses, furin is a desirable target for antiviral compounds [185,186,187]. Accordingly, the inhibition of the P62 cleavage by the irreversible furin inhibitor decanoyl-RVKR-chloromethyl ketone (dec-RVKR-chmk) leads to a significant viral reduction envelope glycoproteins E1 and E2 in infected myoblast cultures. Furthermore, given chloroquine, almost total viral spread and yield were observed [153]. The furin inhibitor phenylacetyl-Arg-Val-Arg-4-amidinobenzylamide and a set of its analogues significantly reduced the viral titer in BHK-21 cells [188].

The endosomal cathepsin B protease was recently confirmed as a proviral host target not only for MLV, Ebola virus, and SARS-CoV infections but also for CHIKV. Cathepsin B mediates the lysosomes-endosomes-fusion and is used by pathogens to enter the cell [119,184]. The antimalaria drug amodiaquine and its primary active metabolite desethyl-amodiaquine (DEAQ) have shown to act as an anti-pathogen against various bacteria toxins and as an inhibitor of multiple viruses (e.g., the CHIKV) by inhibiting the host cathepsin B protease (see Figure 4) [119].

The naturally occurring carboxylic nucleotide aristeromycin (Figure 4) showed potent anti-CHIKV effects, but further usage was limited by its high cytotoxicity [120,189]. This type I S-adenosyl-L-homocysteine hydrolase (SAH) inhibitor was the starting point of a series of 6′-fluorinated aristeromycin analogues (Figure 4). Since both the viral RdRp and the host SAH hydrolase are crucial for the viral RNA capping and replication, the design of dual-target antiviral compounds was performed by Yoon et al. [120]. Surprisingly, the inhibition of the viral RdRp was found to be less important in this new compound series than the SAH inhibition [120]. As already discussed in 4.4.1. nsP1, based on these results, 6′-fluorinated-5′-homoaristeromycin (FHA) and 6′-fluoro-homoneplanocin (FHNA) were synthesised and showed potent anti-CHIKV activity [37]. Interestingly, they seem to target the viral nonstructural protein nsP1 rather than the proviral host factor SAH [38].

##### Kinases

Targeting the proviral host kinases has led to the identification of multiple compounds with interesting antiviral activity. Many viruses modify the host kinases signaling pathways to regulate the cellular environment and to stimulate their replication [190]. The Src family kinases (SFKs), for example, have been identified through kinome profiling as essential proteins for the replication of several viruses, including the CHIKV. Accordingly, the chemical inhibitors of these membrane-associated kinases, dasatinib and the mTORC1/2 inhibitor Torin 1, were able to reduce the viral yields in human fibroblasts (see Figure 4) [121]. Other kinase inhibitor compounds, the CND series (Figure 4), were found through HTS utilising a kinase inhibitory chemical library (BioFocus). A mechanism of action study with this compound series suggested that the inhibition of virus-induced CPE was likely performed by targeting kinases involved in apoptosis [122]. However, their precise target kinase requires further investigation [122].

Another potential proviral host kinase target is the mitogen-activated protein kinase (MAPK) signaling pathway. Berberine (Figure 4), a plant-derived alkaloid, was found with ivermectin and abamectin through a high throughput screening. All three compounds showed good activity against different alphaviruses [123]. Furthermore, the activation of the MAPK during a CHIKV infection and the resulting changes in phosphorylation levels were detected by a human phosphokinase array detected [124]. Berberine treatment decreased the viral titer in HEK-293T cells by reducing this CHIKV induced MAPK activity. Additionally, berberine treatment in CHIKV-infected C57BL6/J led to reduced inflammation in the joint footbed [124]. Recently, berberine was reported to interfere with the virus ability to form a stable cytoplasmic nucleocapsid core (NC)–inhibiting the formation of infectious virus particles [191].

Depending on the host cell, the CHIKV entry occurs via endocytosis or macropinocytosis [98,184]. Macropinocytosis activation occurs when the virus activates signal transduction in the host cell via different cellular proteins such as the phosphatidylinositol-3kinase (PI3K) and the protein kinase C [98]. The inhibition of the AKT-phosphorylation through the interaction with the Pi3-Akt signaling pathway by the anti-leishmaniosis drug miltefosine (Figure 4) led to a reduction of the CHIKV replication in human dermal fibroblasts [125].

The serin/threonine-protein kinase C (PKC) regulates several cellular processes, including cell proliferation and apoptosis [126]. PCK modulators have been reported to inhibit CHIKV replication in vitro. The phorbol ester prostatin (Figure 4) is a potent activator of PCK and showed antiviral activity on different CHIKV strains [126,192]]. The antiviral activity of prostatin, however, was strongly dependent on the used cell type. Potent antiviral activity were reported in BGM and Vero A cells, but the PCK inhibitor showed no antiviral activity in HEL cells [126]. Furthermore, prostatin has been reported to have tumour promoting effects [126]. Analogues of the pan-PCK modulator bryostatin, also potently inhibited the CHIKV replication. Interestingly, when the hydroxyl group on C26, which is vital for the PCK interaction, was capped, the antiviral activity was still found. This suggests an additional PCK-independent mode of action [127,128,193].

Additionally, the cyclin G-associated kinase (GAK) inhibitors with an isothiazolo[4,3-b]pyridine (Figure 4) scaffold showed moderate antiviral activity against the Dengue virus, Ebola virus, and the Chikungunya virus [129].

##### Lyases/transferases

Reducing the polyamine concentration in host cells has negative effects on the viral replication of various RNA viruses as they need it for viral translation and transcription [194]. Intracellular polyamine synthesis relies on a set of different proteins such as ornithine decarboxylase 1 (ODC1) and spermidine/spermine N1-acetyltransferase 1 (SAT1). Depletion of spermidine and spermine by induction of SAT1 led to decreased CHIKV replication [194]. Interestingly, the resistance of this polyamine dependency was found in a CHIKV variant with mutations in the viral nsP1 and nsP4 [195]. The irreversible inhibitor of ODC1, difluoromethylornithine (DFMO, Figure 4), and the SAT1 upregulator, diethylnorspermine (DENSpm, Figure 4), reduced the viral titer in different cell lines. Despite these good in vitro results, DFMO showed a low reduction in viral titer in CHIKV-infected C57BL/6 mice [130].

### 5.6. Cellular Receptor Inhibitors

#### 5.6.1. Inhibitors of Channel-Linked Receptors

Critical steps of the viral replication cycle have been reported to depend on the virus’s ability to manipulate the host cell ionic environment. Consistent with these findings, several viral proteins have been shown to influence cellular ion channel activity [196]. So far, two different ion channel families have been exploited as anti-CHIKV targets–the sodium-potassium ATPase and the chloride channel 1 and 4 (CLIC1, CLIC4) [20,131,132]

**Figure 5:**
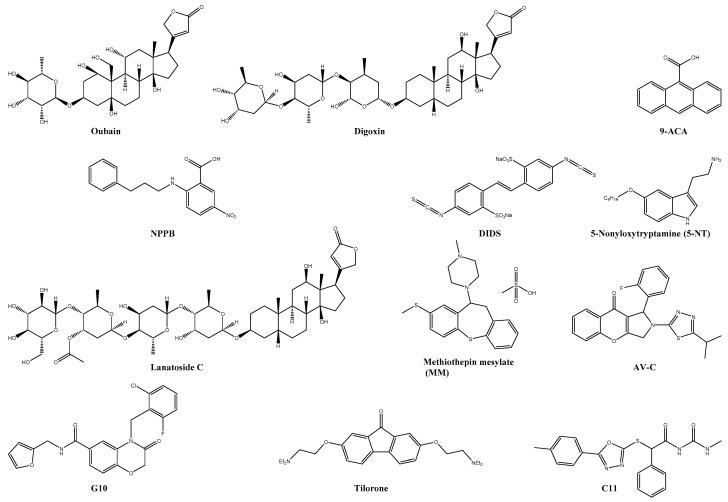
Chemical structure of selected host-targeting compounds. Inhibitors of cellular receptors: oubain, digoxin, lanatoside C, DIDS, 9-ACA, NPPB, 5-nonyloxytryptamine, and methiothepin mesylate. Immunomodulatory agents: tilorone, C11, G10, and AV-C.

A high throughput screening has identified the known sodium-potassium ATPase inhibitor digoxin and its related cardiac glycoside ouabain as a potent CHIKV inhibitor in human cell lines (see Figure 5) [20]. Furthermore, mechanistic studies revealed that digoxin is acting in a post-entry step of the viral replication. Its antiviral effect was reversed when exogenous potassium was added during digoxin treatment, which led to the hypothesis that the CHIKV may require a specific ion balance for its replication. Digoxin-resistant CHIKV mutations carried the V209I mutation in the viral nonstructural protein nsP4, indicating that digoxin may also interact with the viral replication [20]. In addition, another FDA approved cardiac glycoside, lanatoside C (Figure 5), was reported to inhibit a broad spectrum of viruses, including the dengue virus, the Sindbis virus, and the CHIKV [131].

The discovery of CLIC1 and CLIC4 as proviral factors in human cells was performed using the siRNA silencing technique. This resulted in the identification of three chloride channel inhibitors, 4,4′-Diisothiocyanostilbene-2,2′-disulfonic acid (DIDS), 9-Anthracenecarboxylic acid (9-ACA), and 5-Nitro-2-(3-phenylpropylamino)benzoic acid (NPPB, Figure 5). All three compounds showed a significant reduction in CHIKV replication not only in human cell lines (Huh-7) but also in mosquito cells (C6/C3) [132].

#### 5.6.2. Inhibitors of Enzyme-linked Receptors

The serotonin or 5-hydroxytryptamine (5-HT) receptors are primarily G-protein coupled and regulate essential physiological functions and various signaling pathways [134]. The serotonin receptor agonist 5-nonyloxytryptamine (5-NT, Figure 5) has been shown to inhibit CHIKV replication in U2OS cells [134]. Interestingly, also the 5-HT antagonist methiothepin mesylate (MM, Figure 5) was able to inhibit 97 ± 1.0% of the CHIKV replication at 10 µM. A time-of-addition study suggested two different modes of actions: for 5-NT, the inhibition of the uncoating and for MM, the internalisation and membrane hemifusion step [133].

### 5.7. Immunomodulatory Agents

The host immune system and responses, primarily the type I interferon (IFN) signaling, are crucial for controlling and preventing CHIKV infections [197]. Accordingly, many antiviral compounds have shown synergistic effects when given with IFNs [92,104,106,130,166]. Furthermore, the orally available IFN-inducer tilorone dihydrochloride (Figure 5) is known since 1970 for its antiviral activity against SFV in mice [198]. More recently, tilorone was reported to inhibit CHIKV infections in Vero cells [135].

Polyinosinic acid: polycytidylic acid (poly(I:C)), a double-stranded RNA, is another potent IFN inducer and interacts with the toll-like receptor 3 (TLR3) which are expressed in the membrane of B-cells, macrophages, and dendritic cells. The TLR3 receptors are part of the innate immune response and recognize proteins, lipids, carbohydrates and others from invading microorganism. When activated by poly(I:C), they suppress the CHIKV infection by inducing IFNs and other antiviral genes [199]. Pre-treatment of mice with poly(I:C) reduced viral titer in the brain and achieved 100% survivability of the mice [200]. The double-stranded RNA 5′pppRNA is, like poly(I:C), a well-studied adjuvant for enhancing the efficacy of influenza virus vaccines [201,202]. In addition, 5′pppRNA and its analogue M8 inhibited the in vitro and in vivo replication of different viruses (including the CHIKV) by interacting with the retinoic acid-inducible gene I (RIG-I) [203,204,205].

The IFN-inducible protein viperin was also reported to play an essential role in the in vivo infection of the CHIKV and could be an interesting target for antiviral drugs. Mice lacking viperin were reported to develop higher viremia and more severe joint inflammation than infected wild-type mice [206,207]. A novel small molecule (C11, Figure 5) was recently shown to induce IFN secretion from human cells and transcription/translation of interferon-dependent antiviral genes such as viperin. Reverse genetics and a loss-of-function assay suggested the adaptor protein STING for its IFN activation ability. C11 had antiviral effects against the Ross River virus, VEEV, Mayaro virus, O’nyong-nyong virus, and CHIKV [136]. G10 (Figure 5) is another small molecule preventing the replication of various alphaviruses (e.g., VEEV, Sindbis virus, and CHIKV) by indirectly activating the STING protein, which supports the hypothesis of STING as a possible antiviral target [137]. The same research group reported AV-C (Figure 5) as a novel interferon-activating small molecule. AV-C showed inhibitory effects against ZIKV, Dengue virus, and CHIKV infection in THF cells [138].

Further, heparan sulfate mimetics such as pentosan polysulfate and PG545 (pixatimod) have been reported as interesting compounds for alleviating alphavirus-induced disease in vivo. Pentosan polysulfate is an FDA-approved drug against cystitis, whereas pixatimod is currently in a clinical trial to treat advanced cancer and pancreatic adenocarcinoma [139,208,209]. Although pentosan polysulfate treatment did not influence the kinetics of virus infection, it alleviated virus-induced arthritis in C57BL/6 mice [139]. Furthermore, it was recently successfully evaluated in phase II clinical trials for the treatment of RRV-induced arthritic disease (PARA_004, Paradigm BioPharmaceuticals) [210]. Pixatimod treatment also reduced the severity of alphavirus-induced arthritis and showed good antiviral effects against different CHIKV strains [140].

## 6. Undefined Targets

**Figure 6:**
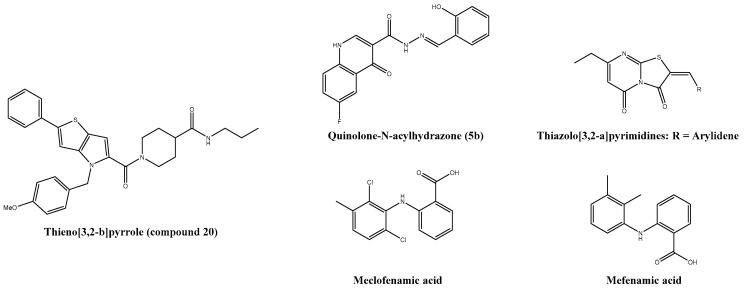
Chemical structure of anti-CHIKV compounds without known target.

Quinolones, like ciprofloxacin and N-acyl hydrazones, were reported as compounds with antibacterial and antiviral properties. Therefore, a series of quinolone-N-acylhydrazone hybrids (Figure 6) were synthesized in a four step synthesis route. Accordingly, they demonstrated antiviral effects against the ZIKV and CHIKV in Vero cells. A mechanism of action study suggests that the compounds act in the early stages and in some post-infection stages of the CHIKV replication cycle. Nevertheless, the precise mode of action remains unknown [211].

The thieno[3,2-b]pyrrole (Figure 6) scaffold was initially found by screening and has been shown to be a potential lead compound. Two structure–activity-relationship studies were made to improve its antiviral activity and its metabolic stability and yielded in the design of the most promising analogue: compound 20. This compound series has been reported to inhibit the expression of viral nsP1, nsP3, capsid, and E2 proteins and to affect the CHIKV life cycle. Additionally, they have been shown to inhibit other alphaviruses such as the O′nyong-nyong and Sindbis virus [212,213].

Recently, Fares et al. reported a new scaffold of a CHIKV inhibitor based on the fusion of uracil and rhodanine pharmacophoric features, which were previously identified as antiviral active (Figure 6) [30,60,214,215]. The best performing analogue, compound 15, had a p-methyl biphenyl tail functionality and may be an interesting lead compound for further drug development [214]]. An overview of compounds without a known target is given in Table 4.

## 7. Conclusions

Due to the severity and chronicity of the Chikungunya fever and the rapid worldwide spread, the CHIKV remains a clinically relevant pathogen. Moreover, the lack of approved antiviral compounds and vaccines against this alphavirus further lightens the crucial development of inhibitors against the Chikungunya virus. A comprehensive overview of several antiviral compounds is given in this review, but most of them are still in the early stage of drug development as their activity against the CHIKV is only tested in in vitro assays. This issue could be overcome by repurposing already approved drugs for the anti-CHIKV treatment. As they already have been intensively investigated for their safety in humans, the clinical evaluation of such drugs could be a fast and safe option for emergency treatment in CHIKV infection outbreaks. The advantage of this approach is illustrated by the fact that out of all compounds discussed in this review only 15 were tested against CHIKV in an animal model, and only one of them (thieno[3,2-b]pyrrole, compound 20) was purposely designed to inhibit the alphavirus. Repurposed drugs such as suramin, favipiravir, sofosbuvir, pimozide, auranofin, PPS, pixatimob, and ribavirin (alone and in combination) showed a reduction of pathological signs in vivo. Furthermore, known inhibitors of proviral host targets (such as TOFA, HS-10, SNX-2112, PACMA31, berberine, and DFMO) reduced the viral titer in the performed in vivo assays. By demonstrating their effects in animal assays, they further confirmed their mode of action to be a valid and potential host target for the development of anti-CHIKV drugs not only in vitro but also in vivo. A broad spectrum of various proviral host targets such as the fatty acid synthase (FASN), calmodulin, IMPDH, TRX, and MAP kinase and more can be therefore considered as interesting targets for future antiviral drug development.

On the other hand, a much more effective and stronger antiviral activity can be expected from compounds directly designed to inhibit the alphavirus. These compounds, such as bis(benzofuran-thiazolidone) (3g), MADTP (9b), CHVB-032, compound 25, compound 8, and compound-A (see Section 4, Virus Targeting Inhibitors) demonstrated already promising antiviral activity in vitro and could be therefore considered for further development and testing.

Another challenge in the development of CHIKV antiviral drugs is its already shown ability to mutate and to develop resistance to antiviral therapy. A combinatory approach of compounds with synergistic effect or the design of an antiviral with multiple targets could diminish this escape mechanism of the virus. Furthermore, recent studies have identified new promising host factors as possible targets for CHIKV inhibitors. Such inhibitors could demonstrate pan-viral inhibitory effects as many viruses use the same replication strategies. However, targeting a crucial host cell factor could also lead to more (serious) side effects due to the manipulation of important biomechanism of the host. More research is required to identify new and safe targets by collecting more detailed information about the CHIKV life cycle. Furthermore, the current absence of a vaccine makes the development of a potent and safe CHIKV inhibitor crucial for the treatment of this severe disease.

## Figures and Tables

**Table 1:** Virus targeting compounds ^a^.

		In Vitro				In Vivo		
Compound ^b^	Viral Target ^c^	EC_50_ (µM) ^d^	CC_50_ (µM)	SI	Cell Line	Efficacy	Mouse Model	Ref.
Arbidol *	E2	12.2 ± 2.2	376	36	MRC5	―	―	[24]
Suramin *	E2	8.8 ± 0.5	>700	>39.1	BHK-21	Reduced viral burden and decreased foot swelling	C57BL/6	[25,26,27]
Picolinic acid	C	60.63% inhibition with 2 mM	n.s.	n.s.	Vero	―	―	[28]
Amantadine *	6K	29.51	n.s.	n.s.	Vero	―	―	[29]
MADTP (9b)	nsP1	1.2 ± 0.009	84 ± 19	70	Vero	―	―	[30,31,32,33]
CHVB-032	nsP1	2.7	>75	n.s.	Vero	―	―	[34,35]
Lobaric acid	nsP1	5.3 ± 0.4	50 ± 1.3	7	Huh-7	―	―	[36]
FHA	nsP1	0.12 ± 0.04	>250	>1000	Vero	―	―	[37,38]
FHNA	nsP1	0.18 ± 0.11	>250	>1000	Vero	―	―	[37,38]
5-IT	nsP1	0.409	>50	n.s.	Vero	―	―	[39]
Compound 25	nsP2	3.2 ± 1.8	101 ± 50	32	Vero	―	―	[40,41,42]
Compound 8	nsP2	1.5	>200	>133.3	BHK-21	―	―	[43]
ID1452-2	nsP2	31	>31	n.s.	HEK293T	―	―	[44]
Novobiocin *	nsP2	20	n.s.	n.s.	Vero	―	―	[45]
Telmisartan *	nsP2	45	n.s.	n.s.	Vero	―	―	[45]
SRI-43750	nsP3	23	>40	n.s.	NHDF	―	―	[46]
Favipiravir *	nsP4	25 ± 3	>636	n.s.	Vero	decreased mortality by >50% and improved disease outcome	AG129	[47]
						Reduced viral replication in joints	C57BL/6J	[48]
NHC	nsP4	0.2 ± 0.1	7.7	n.s.	Vero	―	―	[49,50]
Sofosbuvir *	nsP4	2.7 ± 0.5	402 ± 3 2	149	Huh-7	Reduced viremia and joint pain	SwissWebster	[51]
Compound-A	nsP4	0.54 ± 0.08	3.70 ± 0.32	n.s.	Vero	―	―	[52]

^a^ EC_50_, 50% effective concentration (if no EC_50_ value was reported another readout is presented); CC_50_, 50% cytotoxic concentration; SI, selectivity index; n.s., not specified; ―, not determined; *, repurposed drug. ^b^ If the study reported a compound series/class with anti-CHIKV activity, the antiviral data of the most potent or most representative compound is reported. Only compounds with in vitro or in vivo data are included. ^c^ Compounds are only included in Table 1 if there is enough data about the mode of action. ^d^ If a compound was reported in multiple studies, cell lines, and CHIKV strains, the best activity value with the corresponding cell line is listed.

**Table 2:** Compounds with suggested viral target but without a clear mode of action ^a^.

		In Vitro				In Vivo		
Compound ^b^	Suggested Viral Target	EC_50_ (µM) ^c^	CC_50_ (µM)	SI	Cell Line	Efficacy	Mouse Model	Ref.
IIc	E2	6.5 ± 1	156	22	Vero	―	―	[53]
Bis(benzofuran-thiazolidone)s (3g)		1.5	>200	<133	Vero	―	―	[54]
LQM334	E2	81.1 ± 6.4% viral inhibition	n.s.	n.s.	Vero	―	―	[55]
Micafungin *	E1/E2	17.2 ± 1.08	>100	>5.81	U2OS	―	―	[56]
Doxycycline *	E2/nsP2	15.51 ± 1.62	n.s.	n.s.	Vero	+ ribavirin: Reduction of pathological signs and virus titre	Adult ICR	[57]
AP4	C	10.66 ± 2.25	2172 ± 104	n.s.	Vero	―	―	[58]
EAC	C	4.01 ± 1.96	1657 ± 1109	n.s.	Vero	―	―	[58]
PSU	C	22.91 ± 3.83	2505 ± 0683	n.s.	Vero	―	―	[58]
MBZM-N-IBT	nsP2	38.68	>800	>21	Vero	―	―	[59]
1,3-thiazolidin-4-one (compound 7)	nsP2	0.42	>100	n.s.	Vero	―	―	[60]
peptidomimetic 3a	nsP2	8.76	n.s.	n.s.	Vero	―	―	[61]
PEP-I	nsP2	34	Maximum nontoxic dose is 50 µM	n.s.	BHK-21	―	―	[62]
Nelfinavir *	nsP2	14 ± 1	22 ± 6	1.6	Vero	―	―	[63]

^a^ EC_50_, 50% effective concentration (if no EC_50_ value was reported another readout is presented); CC_50_, 50% cytotoxic concentration; SI, selectivity index; n.s., not specified; ―, not determined; *, repurposed drug. ^b^ If the study reported a compound series/class with anti-CHIKV activity, the antiviral data of the most potent or most representative compound is reported. Only compounds with in vitro or in vivo data are included. ^c^ If a compound was reported in multiple studies, cell lines, and CHIKV strains, the best activity value with the corresponding cell line is listed.

**Table 3:** Host factor targeting compounds ^a^.

		In Vitro				In Vivo		
Compound ^b^	Host Target ^c^	EC_50_ (µM) ^d^	CC_50_ (µM)	SI	Cell Line	Efficacy	Mouse Model	Ref.
Viral Entry and Membrane Fusion								
Chloroquine *	pH	7.0 ± 1.15	>260	37.14	Vero	―	―	[95]
Obatoclax *	Bcl-2/E1	0.03 ± 0.01	20.1 ± 4.8	670	BHK-21	―	―	[96]
Niclosamide *	pH	0.36 ± 0.08	>20	>55.55	U2OS	―	―	[97]
Nitazoxanide *	pH	2.96 ± 0.18	25	8.45	BHK-21	―	―	[97]
EIPA	pH **	Detectable inhibition observed at 0.03 µM	n.s.	n.s.	HSMM	―	―	[98]
Lipid Pathway Inhibitors								
Orlistat *	FASN	0.82	8.67	10.57	HEK293T	―	―	[99,100,101]
Cerulenin *	FASN	3	7.57	2.53	HEK293T	―	―	[99,100]
CAY10566	SCD1				HEK293T	―	―	[100]
TOFA	FASN	0.15	>60	n.s.	HEK293T	Reduction of the viral replication and joint swelling	C57BL/6	[99]
Tivozanib *	FLT4	0.8	8.34	n.s.	HEK293T	―	―	[99]
Pimozide *	calmodulin	0.28	19.18	69.75	HEK293T	Reduction of the viral replication and joint swelling	C57BL/6	[99]
Imipramine *	NPC **	Detectable inhibition observed at 10 µM	n.s.	n.s.	HFF1	―	―	[102]
U18666A	NPC **	Detectable inhibition observed at 0.63 µM	n.s.	n.s.	HFF1	―	―	[102]
LXR-623	LXRβ	2.50	63.30	25.3	HFF	―	―	[103]
Pyrimidine and Purine Synthesis Inhibitors								
Ribavirin *	IMPDH/ (viral) RdRp **	341.1	>30,000	24	Vero	+ doxycycline: Reduction of pathological signs and virus titre	Adult ICR	[57,104]
Merimepodib *	IMPDH	1.8 ± 1.0	27 ± 3	n.s.	Vero	―	―	[105]
6-Azauridine	OMP	0.816	208	204	Vero	―	―	[104]
DD363	DHODH	3.6 ± 0.6	87 ± 7	n.s.	HEK293T	―	―	[106,107]
RYL-634	DHODH	0.26	>2.5	>10	Vero	―	―	[108]
Atovaquone *	DHODH **	<0.75	>11.25	n.s.	Vero	―	―	[109]
Protein Synthesis Inhibitors								
Halofuginone	EPRS	3 log_10_ viral titer reduction at 100 nM	n.s.	n.s.	HFF	―	―	[110]
Harringtonine		0.24	n.s	n.s.	BHK21	―	―	[111]
Sorafenib *	FLT4	0.16	n.s.	n.s.	Vero	―	―	[99,112]
Bortezomib *	UPS **	0.023	0.47	20.6	HeLA	―	―	[113]
SR9009 *	Rev-erb α/β **	100-fold reduction in viral titer at 10 µM	n.s.	n.s.	Huh7	―	―	[114]
Cellular Protein Inhibitors								
Sirtinol	SIRT	>2 log_10_ viral titer reduction at 200 µM	n.s.	n.s.	U2OS	―	―	[115]
Geldanamycin *	HSP90	2.5 log_10_ viral titer reduction at 1.4 µM	>100	n.s.	HEK293T	―	―	[116]
HS-10	HSP90	>2 log_10_ viral reduction in titre with 6.25 µM	>100	n.s.	HEK293T	Reduced viral titer, inflammation, and swelling	SVA129	[116]
SNX-2112	HSP90	>2 log_10_ viral reduction in titre with 6.25 µM	>100	n.s.	HEK293T	Reduced viral titer, inflammation, and swelling	SVA129	[116]
HA15	GRP78	reduction in titre with 25 µM	n.s.	n.s.	Vero	―	―	[117]
16F16	PDI	6.6 ± 0.45	8.9 ± 9.2	1.35	HEK293T	―	―	[118]
PACMA31	PDI	12.1 ± 0.3	12.2 ± 9.7	1.00	HEK293T	Less reduction in footbed swelling and viremia than in auranofin group	C57BL/6	[118]
Auranofin *	TRX	1.0 ± 0.13	1.6 ± 8.6	1.6	HEK293T	reduced footbed swelling and viremia	C57BL/6	[118]
Cellular Enzyme Inhibitors-Hydrolases								
Amodiaquine *	Cathepsin B	18.3	>50	>2	HFF	―	―	[119]
DEAQ	Cathepsin B	17.3	>50	>2.9	HFF	―	―	[119]
Aristeromycin	SAH	0.8	6.3	7.9	Vero	―	―	[120]
6,6′-Difluoroaristeromycin (2c)	SAH	0.13	1.25	>9.6	Vero	―	―	[120]
Cellular Enzyme Inhibitors-Kinases								
Dasatinib *	SFK	>10-fold reduction in viral titer at 20 µM	>50	n.s.	NHDF	―	―	[121]
Torin 1	mTORC1/2	>10-fold reduction in viral titer at 1 µM	>1	n.s.	NHDF	―	―	[121]
CND3514	PKR **	2.2	>50	>22.7	HuH-7	―	―	[122]
Berberine	MAPK	1.8 ± 0.5	>100	>55.6	BHK21	Reduced viremia and disease symptoms	C57BL6/J	[123,124]
Miltefosine *	Pi3-Akt	antiviral activity was observed at 20–40 µM	n.s.	n.s.	hPDF	―	―	[125]
Prostatin	PCK	0.2 ± 0.05	50	n.s.	CRL-2522	―	―	[126]
Bryostatin analogue (4)	PCK	0.8 ± 0.1	>50	n.s.	BGM			[127,128]
Isothiazolo[4,3-b]pyridine (12r)	GAK	antiviral activity was observed <10 µM	n.s.	n.s.	Vero	―	―	[129]
Cellular Enzyme Inhibitors–Lyases/Transferases								
DFMO *	ODC1	200-fold reduction in viral titer at 500 µM	n.s.	n.s.	BHK-21	Low reduction in viral titer	C57BL6/J	[130]
Cellular Receptor Inhibitors–Channel-linked Receptors								
Digoxin *	Na^+^/K^+^ ATPase	0.049	>10	n.s.	U2OS	―	―	[20]
Lanatoside C *	Na^+^/K^+^ ATPase	38.99% reduction of viral titer with 1 µM	>1	n.s.	BHK-21	―	―	[131]
DIDS	CLIC1/4	8-fold reduction in viral titer	n.s.	n.s.	HuH-7	―	―	[132]
9-ACA	CLIC1/4	8-fold reduction in viral titer	n.s.	n.s.	HuH-7	―	―	[132]
NPPB	CLIC1/4	18-fold reduction in viral titer	n.s.	n.s.	HuH-7	―	―	[132]
Cellular Receptor Inhibitors–Enzyme-linked Receptors								
5-NT	5-HT	2.8	>5	n.s.	U2OS	―	―	[133,134]
MM	5-HT	97 ± 1.0% viral reduction 10 µM	>10	n.s.	U2OS	―	―	[133]
Immunomodulatory Agents								
Tilorone *	IFN-inducer	4.2	32	7.6	Vero76	―	―	[135]
C11	STING	EC_90_: 16.44 µM	>50	n.s.	THF	―	―	[136]
G10	STING	IC_90_: 8.01 μM	n.s.	n.s.	THF	―	―	[137]
AV-10	TRIF	IC_90_: 3.54 μM	n.s.	n.s.	THF	―	―	[138]
Pentosan polysulfate *	IL-10 inducer; decreased proinflammatory cytokines levels	―	―	―	―	Reduced disease symptoms	C57BL/6	[139]
Pixatimod *	HPSE **	0.51 ± 0.50	n.s.	n.s.	Vero	Reduced disease symptoms	C57BL/6	[140]

^a^ EC_50_, 50% effective concentration (if no EC_50_ value was reported another readout is presented); CC_50_, 50% cytotoxic concentration; SI, selectivity index; n.s., not specified; ―, not determined; *, repurposed drug; **, suggested target. ^b^ If the study reported a compound series/class with anti-CHIKV activity, the antiviral data of the most potent or most representative compound is reported. Only compounds with in vitro or in vivo data are included. ^c^ The host target is only reported if there is enough data about the mode of action. ^d^ If a compound was reported in multiple studies, cell lines, and CHIKV strains, the best activity value with the corresponding cell line is reported.

**Table 4:** Antiviral compounds without a known target ^a^.

	In Vitro				In Vivo		
Compound ^b^	EC_50_ (µM) ^c^	CC_50_ (µM)	SI	Cell Line	Efficacy	Mouse Model	Ref.
Quinolone-N-acylhydrazone (5b)	1.06 ± 0.08	669 ± 4.33	631.7	Vero	―	―	[211]
Thieno[3,2-b]pyrrole (compound 20)	3-4	>100	n.s.	HEK293T	Good in vivo pharmacokinetics	C57BL/6	[212,213]
Compound 15	42	n.s.	n.s.	MCF-7	―	―	[214]
Mefenamic acid *	13	>100	n.s.	Vero	+ ribavirin: Reduction of viral titre and hypertrophic effects in liver and spleen	Adult ICR	[167]
Meclofenamic acid	18	>100	n.s.	Vero	―	―	[167]
Ivermectin *	0.6 ± 0.1	37.9 ± 7.6	62.4	BHK-21	―	―	[123]
Abamectin *	1.5 ± 0.6	28.2 ± 1.1	19.2	BHK-21	―	―	[123]

^a^ EC_50_, 50% effective concentration; CC_50_, 50% cytotoxic concentration; n.s., not specified; ―, not determined; *, repurposed drug. ^b^ If the study reported a compound series/class with anti-CHIKV activity, the antiviral data of the most potent or most representative compound is reported. Only compounds with in vitro or in vivo data are shown. ^c^ If a compound was reported in multiple studies, cell lines, or if different CHIKV strains have been used, the best activity value with the corresponding cell line is reported.

## References

[1] Robinson M.C. (1955). An epidemic of virus disease in Southern Province, Tanganyika Territory, in 1952-53. I. Clinical features. Trans. R. Soc. Trop. Med. Hyg..

[2] Chretien J.P., Anyamba A., Bedno S.A., Breiman R.F., Sang R., Sergon K., Powers A.M., Onyango C.O., Small J., Tucker C.J. (2007). Drought-associated chikungunya emergence along coastal East Africa. Am. J. Trop. Med. Hyg..

[3] Vazeille M., Moutailler S., Coudrier D., Rousseaux C., Khun H., Huerre M., Thiria J., Bastien Dehecq J.-S., Fontenille D., Schuffenecker I. (2007). Two Chikungunya Isolates from Two Chikungunya Isolates from the Outbreak of La Reunion (Indian Ocean) Exhibit Different Patterns of Infection in the Mosquito, Aedes albopictus. PLoS ONE.

[4] Cassadou S., Boucau S., Petit-Sinturel M., Huc P., Leparc-Goffart I., Ledrans M. (2014). Emergence of chikungunya fever on the French side of Saint Martin island, October to December 2013. Eurosurveillance.

[5] Zeller H., Van Bortel W., Sudre B. (2015). Chikungunya: Its History in Africa and Asia and Its Spread to New Regions in 2013–2014. J. Infect. Dis..

[6] Rezza G., Nicoletti L., Angelini R., Romi R., Finarelli A.C., Panning M., Cordioli P., Fortuna C., Boros S., Magurano F. (2007). CHIKV study group. Infection with chikungunya virus in Italy: An outbreak in a temperate region. Lancet.

[7] Venturi G., Di Luca M., Fortuna C., Remoli M.E., Riccardo F., Severini F., Toma L., Del Manso M., Benedetti E., Caporali M.G. (2017). Detection of a chikungunya outbreak in central Italy, August to September 2017. Eurosurveillance.

[8] Delisle E., Rousseau C., Broche B., Leparc-Goffart I., L’ambert G., Cochet A., Prat C., Foulongne V., Ferré J.B., Catelinois O. (2015). Chikungunya outbreak in Montpellier, France, September to October 2014. Eurosurveillance.

[9] Calba C., Guerbois-Galla M., Franke F., Jeannin C., Auzet-Caillaud M., Grard G., Pigaglio L., Decoppet A., Weicherding J., Savaill M.C. (2017). Preliminary report of an autochthonous chikungunya outbreak in France, July to September 2017. Eurosurveillance.

[10] Campbell L.P., Luther C., Moo-Llanes D., Ramsey J.M., Danis-Lozano R., Peterson A.T. (2015). Climate change influences on global distributions of dengue and chikungunya virus vectors. Philos. Trans. R. Soc. B Biol. Sci..

[11] WHO Media Centre Chikungunya—Key Facts. https://www.who.int/news-room/fact-sheets/detail/chikungunya.

[12] Thiberville S.-D., Moyen N., Dupuis-Maguiraga L., Nougairede A., Gould E.A., Roques P., De Lamballerie X. (2013). Chikungunya fever: Epidemiology, clinical syndrome, pathogenesis and therapy. Antivir. Res..

[13] Borgherini G., Poubeau P., Jossaume A., Gouix A., Cotte L., Michault A., Arvin-Berod C., Paganin F. (2008). Persistent arthralgia associated with chikungunya virus: A study of 88 adult patients on reunion island. Clin. Infect. Dis..

[14] Lebrun G., Chadda K., Reboux A.H., Martinet O., Gaüzère B.A. (2009). Guillain-Barré Syndrome after Chikungunya Infection. Emerg. Infect. Dis..

[15] Kennedy Amaral Pereira J., Schoen R.T. (2017). Management of chikungunya arthritis. Clin. Rheumatol..

[16] Kielian M., Chanel-Vos C., Liao M. (2010). Alphavirus Entry and Membrane Fusion. Viruses.

[17] Voss J.E., Vaney M.-C., Duquerroy S., Vonrhein C., Girard-Blanc C., Crublet E., Thompson A., Bricogne G., Rey F.A. (2010). Glycoprotein organization of Chikungunya virus particles revealed by X-ray crystallography. Nature.

[18] Solignat M., Gay B., Higgs S., Briant L., Devaux C. (2009). Replication cycle of chikungunya: A re-emerging arbovirus. Virology.

[19] Van Der Heijden M.W., Bol J.F. (2002). Composition of alphavirus-like replication complexes: Involvement of virus and host encoded proteins. Arch Virol.

[20] Ashbrook A.W., Lentscher A.J., Zamora P.F., Silva L.A., May N.A., Bauer J.A., Morrison T.E., Dermody T.S. (2016). Antagonism of the Sodium-Potassium ATPase Impairs Chikungunya Virus Infection. MBio.

[21] Abdelnabi R., Neyts J., Delang L. (2015). Towards antivirals against chikungunya virus. Antivir. Res..

[22] Jose J., Snyder J.E., Kuhn R.J., Snyder E.J., Kuhn R.J. (2009). A structural and functional perspective of alphavirus replication and assembly. Futur. Med..

[23] Strauss J.H., Strauss E.G. (1994). The alphaviruses: Gene expression, replication, and evolution. Microbiol. Rev.

[24] Delogu I., Pastorino B., Baronti C., Nougairède A., Bonnet E., de Lamballerie X. (2011). In vitro antiviral activity of arbidol against Chikungunya virus and characteristics of a selected resistant mutant. Antivir. Res..

[25] Ho Y.-J., Wang Y.-M., Lu J., Wu T.-Y., Lin L.-I., Kuo S.-C., Lin C.-C. (2015). Suramin Inhibits Chikungunya Virus Entry and Transmission. PLoS ONE.

[26] Kuo S.-C., Wang Y.-M., Ho Y.-J., Chang T.-Y., Lai Z.-Z., Tsui P.-Y., Wu T.-Y., Lin C.-C. (2016). Suramin treatment reduces chikungunya pathogenesis in mice. Antivir. Res..

[27] Albulescu I.C., Van Hoolwerff M., Wolters L.A., Bottaro E., Nastruzzi C., Yang S.C., Tsay S.-C., Hwu J.R., Snijder E.J., Van Hemert M.J. (2015). Suramin inhibits chikungunya virus replication through multiple mechanisms. Antivir. Res..

[28] Sharma R., Fatma B., Saha A., Bajpai S., Sistla S., Dash P.K., Parida M., Kumar P., Tomar S. (2016). Inhibition of chikungunya virus by picolinate that targets viral capsid protein. Virology.

[29] Dey D., Siddiqui S.I., Mamidi P., Ghosh S., Kumar C.S., Chattopadhyay S., Ghosh S., Banerjee M. (2019). The effect of amantadine on an ion channel protein from Chikungunya virus. PLoS Negl. Trop. Dis..

[30] Gigante A., Canela M.-D.D., Delang L., Priego E.-M.M., Camarasa M.-J.J., Querat G., Neyts J., Leyssen P., Pérez-Pérez M.-J.J. (2014). Identification of [1,2,3]triazolo[4,5-d]pyrimidin-7(6H)-ones as novel inhibitors of Chikungunya virus replication. J. Med. Chem..

[31] Delang L., Li C., Tas A., Quérat G., Albulescu I.C., De Burghgraeve T., Guerrero N.A.S., Gigante A., Piorkowski G., Decroly E. (2016). The viral capping enzyme nsP1: A novel target for the inhibition of chikungunya virus infection. Sci. Rep..

[32] Gigante A., Gómez-SanJuan A., Delang L., Li C., Bueno O., Gamo A.-M., Priego E.-M. (2017). Antiviral activity of [1,2,3]triazolo[4,5-d]pyrimidin-7(6H)-ones against chikungunya virus targeting the viral capping nsP1. Antivir. Res..

[33] Gómez-SanJuan A., Gamo A.-M., Delang L., Pérez-Sánchez A., Amrun S.N., Abdelnabi R., Jacobs S., Priego E.-M., Camarasa M.-J., Jochmans D. (2018). Inhibition of the Replication of Different Strains of Chikungunya Virus by 3-Aryl-[1,2,3]triazolo[4,5- d]pyrimidin-7(6 H)-ones. ACS Infect. Dis..

[34] Moesslacher J., Battisti V., Delang L., Neyts J., Abdelnabi R., Pürstinger G., Urban E., Langer T. (2020). Identification of 2-(4-(Phenylsulfonyl)piperazine-1-yl)pyrimidine Analogues as Novel Inhibitors of Chikungunya Virus. ACS Med. Chem. Lett..

[35] Abdelnabi R., Kovacikova K., Moesslacher J., Donckers K., Battisti V., Leyssen P., Langer T., Puerstinger G., Quérat G., Li C. (2020). Novel Class of Chikungunya Virus Small Molecule Inhibitors That Targets the Viral Capping Machinery. Antimicrob. Agents Chemother..

[36] Feibelman K.M., Fuller B.P., Li L., LaBarbera D.V., Geiss B.J. (2018). Identification of small molecule inhibitors of the Chikungunya virus nsP1 RNA capping enzyme. Antivir. Res..

[37] Shin Y.S., Jarhad D.B., Jang M.H., Kovacikova K., Kim G., Yoon J., Kim H.R., Hyun Y.E., Tipnis A.S., Chang T.S. (2020). Identification of 6′-β-fluoro-homoaristeromycin as a potent inhibitor of chikungunya virus replication. Eur. J. Med. Chem..

[38] Kovacikova K., Morren B.M., Tas A., Albulescu I.C., Van Rijswijk R., Jarhad D.B., Shin Y.S., Jang M.H., Kim G., Lee H.W. (2020). 6′-β-Fluoro-Homoaristeromycin and 6′-Fluoro-Homoneplanocin A Are Potent Inhibitors of Chikungunya Virus Replication through Their Direct Effect on Viral Nonstructural Protein 1. Antimicrob. Agents Chemother..

[39] Mudgal R., Mahajan S., Tomar S. (2020). Inhibition of Chikungunya virus by an adenosine analog targeting the SAM-dependent nsP1 methyltransferase. FEBS Lett..

[40] Bassetto M., De Burghgraeve T., Delang L., Massarotti A., Coluccia A., Zonta N., Gatti V., Colombano G., Sorba G., Silvestri R. (2013). Computer-aided identification, design and synthesis of a novel series of compounds with selective antiviral activity against chikungunya virus. Antivir. Res..

[41] Tardugno R., Giancotti G., De Burghgraeve T., Delang L., Neyts J., Leyssen P., Brancale A., Bassetto M. (2018). Design, synthesis and evaluation against Chikungunya virus of novel small-molecule antiviral agents. Bioorg. Med. Chem..

[42] Giancotti G., Cancellieri M., Balboni A., Giustiniano M., Novellino E., Delang L., Neyts J., Leyssen P., Brancale A., Bassetto M. (2018). Rational modifications on a benzylidene-acrylohydrazide antiviral scaffold, synthesis and evaluation of bioactivity against Chikungunya virus. Eur. J. Med. Chem..

[43] Das P.K., Puusepp L., Varghese F.S., Utt A., Ahola T., Kananovich D.G., Lopp M., Merits A., Karelson M. (2016). Design and Validation of Novel Chikungunya Virus Protease Inhibitors. Antimicrob. Agents Chemother..

[44] Lucas-Hourani M., Lupan A., Desprès P., Thoret S., Pamlard O., Dubois J., Guillou C., Tangy F., Vidalain P.-O., Munier-Lehmann H. (2013). A phenotypic assay to identify Chikungunya virus inhibitors targeting the nonstructural protein nsP2. J. Biomol. Screen..

[45] Tripathi P.K., Soni A., Singh Yadav S.P., Kumar A., Gaurav N., Raghavendhar S., Sharma P., Sunil S., Ashish, Jayaram B. (2020). Evaluation of novobiocin and telmisartan for anti-CHIKV activity. Virology.

[46] Zhang S., Garzan A., Haese Id N., Bostwick R., Martinez-Gzegozewska Y., Rasmussen L., Streblow D.N., Haise M.T., Pathak A.K., Augelli-Szafran C.E. (2021). Pyrimidone inhibitors targeting Chikungunya Virus nsP3 macrodomain by fragment-based drug design. PLoS ONE.

[47] Delang L., Segura Guerrero N., Tas A., Quérat G., Pastorino B., Froeyen M., Dallmeier K., Jochmans D., Herdewijn P., Bello F. (2014). Mutations in the chikungunya virus non-structural proteins cause resistance to favipiravir (T-705), a broad-spectrum antiviral. J. Antimicrob. Chemother..

[48] Abdelnabi R., Jochmans D., Verbeken E., Neyts J., Delang L. (2017). Antiviral treatment efficiently inhibits chikungunya virus infection in the joints of mice during the acute but not during the chronic phase of the infection. Antivir. Res..

[49] Ehteshami M., Tao S., Zandi K., Hsiao H.-M., Jiang Y., Hammond E., Amblard F., Russell O.O., Merits A., Schinazi R.F. (2017). Characterization of β-d-N4-Hydroxycytidine as a Novel Inhibitor of Chikungunya Virus. Antimicrob. Agents Chemother..

[50] Urakova N., Kuznetsova V., Crossman D.K., Sokratian A., Guthrie D.B., Kolykhalov A.A., Lockwood M.A., Natchus M.G., Crowley M.R., Painter G.R. (2017). β-D-N4- Hydroxycytidine is a potent anti-alphavirus compound that induces a high level of mutations in the viral genome. J. Virol..

[51] Ferreira A.C., Reis P.A., de Freitas C.S., Sacramento C.Q., Hoelz L.V.B., Bastos M.M., Mattos M., Rocha N., de Azevedo Quintanilha I.G., da Silva Gouveia Pedrosa C. (2019). Beyond members of the Flaviviridae family, sofosbuvir also inhibits chikungunya virus replication. Antimicrob. Agents Chemother..

[52] Wada Y., Orba Y., Sasaki M., Kobayashi S., Carr M.J., Nobori H., Sato A., Hall W.W., Sawa H. (2017). Discovery of a novel antiviral agent targeting the nonstructural protein 4 (nsP4) of chikungunya virus. Virology. Virology.

[53] Scuotto M., Abdelnabi R., Collarile S., Schiraldi C., Delang L., Massa A., Ferla S., Brancale A., Leyssen P., Neyts J. (2017). Discovery of novel multi-target indole-based derivatives as potent and selective inhibitors of chikungunya virus replication. Bioorg. Med. Chem..

[54] Hwu J.R., Gupta N.K., Tsay S.C., Huang W.C., Albulescu I.C., Kovacikova K., van Hemert M.J. (2017). Bis(benzofuran-thiazolidinone)s and bis(benzofuran-thiazinanone)s as inhibiting agents for chikungunya virus. Antivir. Res..

[55] Passos G.F.S., Gomes M.G.M., de Aquino T.M., de Araújo-Júnior J.X., de Souza S.J.M., Cavalcante J.P.M., Dos Santos E.C., Bassi Ê.J., da Silva-Júnior E.F. (2020). Computer-Aided Design, Synthesis, and Antiviral Evaluation of Novel Acrylamides as Potential Inhibitors of E3-E2-E1 Glycoproteins Complex from Chikungunya Virus. Pharmaceuticals.

[56] Ho Y.J., Liu F.C., Yeh C.T., Yang C.M., Lin C.C., Lin T.Y., Hsieh P.S., Hu M.K., Gong Z., Lu J.W. (2018). Micafungin is a novel anti-viral agent of chikungunya virus through multiple mechanisms. Antivir. Res..

[57] Rothan H.A., Bahrani H., Mohamed Z., Teoh T.C., Shankar E.M., Rahman N.A., Yusof R. (2015). A Combination of Doxycycline and Ribavirin Alleviated Chikungunya Infection. PLoS ONE.

[58] Fatma B., Kumar R., Singh V.A., Nehul S., Sharma R., Kesari P., Kuhn R.J., Tomar S. (2020). Alphavirus capsid protease inhibitors as potential antiviral agents for Chikungunya infection. Antivir. Res..

[59] Mishra P., Kumar A., Mamidi P., Kumar S., Basantray I., Saswat T., Das I., Nayak T.K., Chattopadhyay S., Subudhi B.B. (2016). Inhibition of Chikungunya Virus Replication by 1-[(2-Methylbenzimidazol-1-yl) Methyl]-2-Oxo-Indolin-3-ylidene] Amino] Thiourea (MBZM-N-IBT). Sci. Rep..

[60] Singh Jadav S., Sinha B.N., Hilgenfeld R., Pastorino B., De Lamballerie X., Jayaprakash V. (2015). Thiazolidone derivatives as inhibitors of chikungunya virus. Eur. J. Med. Chem..

[61] El-labbad E.M., Ismail M.A.H., Abou Ei Ella D.A., Ahmed M., Wang F., Barakat K.H., Abouzid K.A.M. (2015). Discovery of Novel Peptidomimetics as Irreversible CHIKV NsP2 Protease Inhibitors Using Quantum Mechanical-Based Ligand Descriptors. Chem. Biol. Drug Des..

[62] Singh H., Mudgal R., Narwal M., Kaur R., Singh V.A., Malik A., Chaudhary M., Tomar S. (2018). Chikungunya virus inhibition by peptidomimetic inhibitors targeting virus-specific cysteine protease. Biochimie.

[63] Bhakat S., Delang L., Kaptein S., Neyts J., Leyssen P., Jayaprakash V. (2015). Reaching beyond HIV/HCV: Nelfinavir as a potential starting point for broad-spectrum protease inhibitors against dengue and chikungunya virus. RSC Adv..

[64] Di Mola A., Peduto A., La Gatta A., Delang L., Pastorino B., Neyts J., Leyssen P., de Rosa M., Filosa R. (2014). Structure–activity relationship study of arbidol derivatives as inhibitors of chikungunya virus replication. Bioorg. Med. Chem..

[65] Henß L., Beck S., Weidner T., Biedenkopf N., Sliva K., Weber C., Becker S., Schnierle B.S. (2016). Suramin is a potent inhibitor of Chikungunya and Ebola virus cell entry. Virol. J..

[66] Albulescu I.C., White-Scholten L., Tas A., Hoornweg T.E., Ferla S., Kovacikova K., Smit J.M., Brancale A., Snijder E.J., van Hemert M.J. (2020). Suramin Inhibits Chikungunya Virus Replication by Interacting with Virions and Blocking the Early Steps of Infection. Viruses.

[67] Lu J.W., Hsieh P.S., Lin C.C., Hu M.K., Huang S.M., Wang Y.M., Liang C.Y., Gong Z., Ho Y.J. (2017). Synergistic effects of combination treatment using EGCG and suramin against the chikungunya virus. Biochem. Biophys. Res. Commun..

[68] Weber C., Sliva K., von Rhein C., Kümmerer B.M., Schnierle B.S. (2015). The green tea catechin, epigallocatechin gallate inhibits chikungunya virus infection. Antivir. Res..

[69] Loke R.H.T., Anderson M.G., Coleman J.C., Murray-Lyon I.M., Tsiquaye K.N., Zuckerman A.J. (1987). Suramin treatment for chronic active hepatitis B—toxic and ineffective. J. Med. Virol..

[70] Kaplan L.D., Wolfe P.R., Volberding P.A., Feorino P., Abrams D.I., Levy J.A., Wong R., Kaufman L., Gottlieb M.S. (1987). Lack of response to suramin in patients with AIDS and AIDS-related complex. Am. J. Med..

[71] Agarwal G., Gupta S., Gabrani R., Gupta A., Chaudhary V.K., Gupta V. (2019). Virtual screening of inhibitors against Envelope glycoprotein of Chikungunya Virus: A drug repositioning approach. Bioinformation.

[72] Fernandez-Pol J.A., Fernandez-Pol S. (2010). Method to Control Dengue Viruses in Humans by Picolinic Acid and Derivates Thereof 2010. U.S. Patent.

[73] Kim H.Y., Kuhn R.J., Patkar C., Warrier R., Cushman M. (2007). Synthesis of dioxane-based antiviral agents and evaluation of their biological activities as inhibitors of Sindbis virus replication. Bioorganic Med. Chem..

[74] Aggarwal M., Tapas S., Preeti, Siwach A., Kumar P., Kuhn R.J., Tomar S. (2012). Crystal Structure of Aura Virus Capsid Protease and Its Complex with Dioxane: New Insights into Capsid-Glycoprotein Molecular Contacts. PLoS ONE.

[75] Aggarwal M., Kaur R., Saha A., Mudgal R., Yadav R., Dash P.K., Parida M., Kumar P., Tomar S. (2017). Evaluation of antiviral activity of piperazine against Chikungunya virus targeting hydrophobic pocket of alphavirus capsid protein. Antivir. Res..

[76] Sharma R., Kesari P., Kumar P., Tomar S. (2018). Structure-function insights into chikungunya virus capsid protein: Small molecules targeting capsid hydrophobic pocket. Virology.

[77] Jing X., Ma C., Ohigashi Y., Oliveira A.F., Jardetzky S.T., Pinto H.L., Lamb A.R. (2008). Functional studies indicate amantadine binds to the pore of the influenza A virus M2 proton-selective ion channel. Proc. Natl. Acad. Sci. USA.

[78] Cheung J., Frankling M., Mancia F., Rudolph M., Cassidy M., Gary E., Burshteyn F., Love J. RCSB PDB—3TRK: Structure of the Chikungunya Virus nsP2 Protease; 2011. https://www.rcsb.org/structure/3TRK.

[79] Meena M.K., Kumar D., Kumari K., Kaushik N.K., Kumar R.V., Bahadur I., Vodwal L., Singh P. (2021). Promising inhibitors of nsp2 of CHIKV using molecular docking and temperature-dependent molecular dynamics simulations. J. Biomol. Struct. Dyn..

[80] Montes-Grajales D., Puerta-Guardo H., Espinosa D.A., Harris E., Caicedo-Torres W., Olivero-Verbel J., Martínez-Romero E. (2020). In silico drug repurposing for the identification of potential candidate molecules against arboviruses infection. Antivir. Res..

[81] Khan N., Bhat R., Patel A.K., Ray P. (2021). Discovery of small molecule inhibitors of chikungunya virus proteins (nsP2 and E1) using in silico approaches. J. Biomol. Struct. Dyn..

[82] Kumar P., Kumar D., Giri R. (2019). Targeting the nsp2 cysteine protease of Chikungunya virus using FDA approved library and selected Cysteine protease inhibitors. Pathogens.

[83] Jain J., Kumari A., Somvanshi P., Grover A., Pai S., Sunil S. (2017). In silico analysis of natural compounds targeting structural and nonstructural proteins of chikungunya virus. F1000Research.

[84] Agarwal T., Asthana S., Bissoyi A. (2015). Molecular modeling and docking study to elucidate novel chikungunya virus nsP2 protease inhibitors. Indian J. Pharm. Sci..

[85] Nguyen P.T.V., Yu H., Keller P.A. (2015). Identification of chikungunya virus nsP2 protease inhibitors using structure-base approaches. J. Mol. Graph. Model..

[86] Bora L. (2012). Homology Modeling and Docking to Potential Novel Inhibitor for Chikungunya (37997) Protein nsP2 Protease. J Proteomics Bioinform.

[87] Singh K.D., Kirubakaran P., Nagarajan S., Sakkiah S., Muthusamy K., Velmurgan D., Jeyakanthan J. (2012). Homology modeling, molecular dynamics, e-pharmacophore mapping and docking study of Chikungunya virus nsP2 protease. J. Mol. Model..

[88] Dhindwal S., Kesari P., Singh H., Kumar P., Tomar S., Sarma R.H. (2017). Conformer and pharmacophore based identification of peptidomimetic inhibitors of chikungunya virus nsP2 protease. J. Biomol. Struct. Dyn..

[89] Nguyen P.T.V., Yu H., Keller P.A. (2014). Discovery of in silico hits targeting the nsP3 macro domain of chikungunya virus. J. Mol. Model..

[90] Abu Bakar F., Ng L. (2018). Nonstructural Proteins of Alphavirus—Potential Targets for Drug Development. Viruses.

[91] Abdelnabi R., de Morais A.T.S., Leyssen P., Imbert I., Beaucourt S., Blanc H., Froeyen M., Vignuzzi M., Canard B., Neyts J. (2017). Understanding the Mechanism of the Broad-Spectrum Antiviral Activity of Favipiravir (T-705): Key Role of the F1 Motif of the Viral Polymerase. J. Virol..

[92] Franco E.J., Rodriquez J.L., Pomeroy J.J., Hanrahan K.C., Brown A.N. (2018). The effectiveness of antiviral agents with broad-spectrum activity against chikungunya virus varies between host cell lines. Antivir. Chem. Chemother..

[93] Julander J.G., Dagley A., Gebre M., Komeno T., Nakajima N., Smee D.F., Furuta Y. (2020). Strain-dependent disease and response to favipiravir treatment in mice infected with Chikungunya virus. Antivir. Res..

[94] Kumar S.P., Kapopara R.G., Patni M.I., Pandya H.A., Jasrai Y.T., Patel S.K. (2012). Exploring the polymerase activity of chikungunya viral non structural protein 4 (nsP4) using molecular modeling, e-pharmacophore and docking studies. Int. J. Pharm. Life Sci..

[95] Khan M., Santhosh S.R., Tiwari M., Lakshmana Roa P.V., Parida M. (2010). Assessment of in vitro prophylactic and therapeutic efficacy of chloroquine against chikungunya virus in Vero cells. J. Med. Virol..

[96] Varghese F.S., Rausalu K., Hakanen M., Saul S., Kümmerer B.M., Susi P., Merits A., Ahola T. (2017). Obatoclax Inhibits Alphavirus Membrane Fusion by Neutralizing the Acidic Environment of Endocytic Compartments. Antimicrob. Agents Chemother..

[97] Wang Y.-M., Lu J.-W., Lin C.-C., Chin Y.-F., Wu T.-Y., Lin L.-I., Lai Z.-Z., Kuo S.-C., Ho Y.-J. (2016). Antiviral activities of niclosamide and nitazoxanide against chikungunya virus entry and transmission. Antivir. Res..

[98] Hua C., Lee R., Hussain K.M., Jang J., Chuid H. (2019). Macropinocytosis dependent entry of Chikungunya virus into human muscle cells. PLoS Negl. Trop. Dis..

[99] Karlas A., Berre S., Couderc T., Varjak M., Braun P., Meyer M., Gangneux N., Karo-Astover L., Weege F., Raftery M. (2016). A human genome-wide loss-of-function screen identifies effective chikungunya antiviral drugs. Nat. Commun..

[100] Bakhache W., Neyret A., McKellar J., Clop C., Bernard E., Weger-Lucarelli J., Briant L. (2019). Fatty acid synthase and stearoyl-CoA desaturase-1 are conserved druggable cofactors of Old World Alphavirus genome replication. Antivir. Res..

[101] Hitakarun A., Khongwichit S., Wikan N., Roytrakul S., Yoksan S., Rajakam S., Davidson A.D., Smith D.R. (2020). Evaluation of the antiviral activity of orlistat (tetrahydrolipstatin) against dengue virus, Japanese encephalitis virus, Zika virus and chikungunya virus. Sci. Rep..

[102] Wichit S., Hamel R., Bernard E., Talignani L., Diop F., Ferraris P., Liegeois F., Ekchariyawat P., Luplertlop N., Surasombatpattana P. (2017). Imipramine Inhibits Chikungunya Virus Replication in Human Skin Fibroblasts through Interference with Intracellular Cholesterol Trafficking. Sci. Rep..

[103] Hwang J., Wang Y., Fikrig E. (2019). Inhibition of chikungunya virus replication in primary human fibroblasts by liver X receptor agonist. Antimicrob. Agents Chemother..

[104] Briolant S., Garin D., Scaramozzino N., Jouan A., Crance J.M. (2004). In vitro inhibition of Chikungunya and Semliki Forest viruses replication by antiviral compounds: Synergistic effect of interferon-alpha and ribavirin combination. Antivir. Res..

[105] Tong X., Smith J., Bukreyeva N., Koma T., Manning J.T., Kalkeri R., Kwong A.D., Paessler S. (2018). Merimepodib, an IMPDH inhibitor, suppresses replication of Zika virus and other emerging viral pathogens. Antivir. Res..

[106] Lucas-Hourani M., Dauzonne D., Jorda P., Lle Cousin G., Lupan A., Helynck O., Gory Caignard G., Ve Janvier G., Naë Lle André -Leroux G., Khiar S. (2013). Inhibition of Pyrimidine Biosynthesis Pathway Suppresses Viral Growth through Innate Immunity. PLoS Pathog..

[107] Lucas-Hourani M., Dauzonne D., Munier-Lehmann H., Khiar S., Nisole S., Dairou J., Helynck O., Afonso P.V., Tangy F., Vidalain P.O. (2017). Original chemical series of pyrimidine biosynthesis inhibitors that boost the antiviral interferon response. Antimicrob. Agents Chemother..

[108] Yang Y., Cao L., Gao H., Wu Y., Wang Y., Fang F., Lan T., Lou Z., Rao Y. (2019). Discovery, Optimization, and Target Identification of Novel Potent Broad-Spectrum Antiviral Inhibitors. J. Med. Chem..

[109] Cifuentes Kottkamp A., De Jesus E., Grande R., Brown J.A., Jacobs A.R., Lim J.K., Stapleford K.A. (2019). Atovaquone Inhibits Arbovirus Replication through the Depletion of Intracellular Nucleotides. J. Virol..

[110] Hwang J., Jiang A., Fikrig E. (2019). A potent prolyl tRNA synthetase inhibitor antagonizes Chikungunya and Dengue viruses. Antivir. Res..

[111] Kaur P., Thiruchelvan M., Lee R.C.H., Chen H., Chen K.C., Ng M.L., Chu J.J.H. (2013). Inhibition of chikungunya virus replication by harringtonine, a novel antiviral that suppresses viral protein expression. Antimicrob. Agents Chemother..

[112] Lundberg L., Brahms A., Hooper I., Carey B., Lin S.C., Dahal B., Narayanan A., Kehn-Hall K. (2018). Repurposed FDA-Approved drug sorafenib reduces replication of Venezuelan equine encephalitis virus and other alphaviruses. Antivir. Res..

[113] Kaur P., Lello L.S., Utt A., Dutta S.K., Merits A., Chu J.J.H. (2020). Bortezomib inhibits chikungunya virus replication by interfering with viral protein synthesis. PLoS Negl. Trop. Dis..

[114] Hwang J., Jiang A., Fikrig E. (2018). Rev-erb Agonist Inhibits Chikungunya and O’nyong’nyong Virus Replication. Open Forum Infect. Dis..

[115] Hackett B.A., Dittmar M., Segrist E., Pittenger N., To J., Griesman T., Gordesky-Gold B., Schultz D.C., Cherry S. (2019). Sirtuin inhibitors are broadly antiviral against arboviruses. MBio.

[116] Rathore A.P.S., Haystead T., Das P.K., Merits A., Ng M.L., Vasudevan S.G. (2014). Chikungunya virus nsP3 & nsP4 interacts with HSP-90 to promote virus replication: HSP-90 inhibitors reduce CHIKV infection and inflammation in vivo. Antivir. Res..

[117] Barrera M.D., Callahan V., Akhrymuk I., Bhalla N., Zhou W., Campbell C., Narayanan A., Kehn-hall K. (2021). Proteomic discovery of veev e2-host partner interactions identifies grp78 inhibitor ha15 as a potential therapeutic for alphavirus infections. Pathogens.

[118] Langsjoen R.M., Auguste A.J., Rossi S.L., Roundy C.M., Penate H.N., Kastis M., Schnizlein M.K., Le K.C., Haller S.L., Chen R. (2017). Host oxidative folding pathways offer novel anti-chikungunya virus drug targets with broad spectrum potential. Antivir. Res..

[119] Zilbermintz L., Leonardi W., Jeong S.Y., Sjodt M., McComb R., Ho C.L.C., Retterer C., Gharaibeh D., Zamani R., Soloveva V. (2015). Identification of agents effective against multiple toxins and viruses by host-oriented cell targeting. Sci. Rep..

[120] Yoon J.S., Kim G., Jarhad D.B., Kim H.R., Shin Y.S., Qu S., Sahu P.K., Kim H.O., Lee H.W., Wang S.B. (2019). Design, Synthesis, and Anti-RNA Virus Activity of 6′-Fluorinated-Aristeromycin Analogues. J. Med. Chem..

[121] Broeckel R., Sarkar S., May N.A., Totonchy J., Kreklywich C.N., Smith P., Graves L., DeFilippis V.R., Heise M.T., Morrison T.E. (2019). Src family kinase inhibitors block translation of alphavirus subgenomic mRNAs. Antimicrob. Agents Chemother..

[122] Cruz D.J.M., Bonotto R.M., Gomes R.G.B., da Silva C.T., Taniguchi J.B., No J.H., Lombardot B., Schwartz O., Hansen M.A.E., Freitas-Junior L.H. (2013). Identification of Novel Compounds Inhibiting Chikungunya Virus-Induced Cell Death by High Throughput Screening of a Kinase Inhibitor Library. PLoS Negl. Trop. Dis..

[123] Varghese F.S., Kaukinen P., Gläsker S., Bespalov M., Hanski L., Wennerberg K., Kümmerer B.M., Ahola T. (2016). Discovery of berberine, abamectin and ivermectin as antivirals against chikungunya and other alphaviruses. Antivir. Res..

[124] Varghese F.S., Thaa B., Amrun S.N., Simarmata D., Rausalu K., Nyman T.A., Merits A., McInerney G.M., Ng L.F.P., Ahola T. (2016). The Antiviral Alkaloid Berberine Reduces Chikungunya Virus-Induced Mitogen-Activated Protein Kinase Signaling. J. Virol..

[125] Sharma A., Bhomia M., Yeh T.-J.J., Singh J., Maheshwari R.K. (2018). Miltefosine inhibits Chikungunya virus replication in human primary dermal fibroblasts. F1000Research.

[126] Abdelnabi R., Amrun S.N., Ng L.F.P., Leyssen P., Neyts J., Delang L. (2017). Protein kinases C as potential host targets for the inhibition of chikungunya virus replication. Antivir. Res..

[127] Staveness D., Abdelnabi R., Schrier A.J., Loy B.A., Verma V.A., DeChristopher B.A., Near K.E., Neyts J., Delang L., Leyssen P. (2016). Simplified Bryostatin Analogues Protect Cells from Chikungunya Virus-Induced Cell Death. J. Nat. Prod..

[128] Staveness D., Abdelnabi R., Near K.E., Nakagawa Y., Neyts J., Delang L., Leyssen P., Wender P.A. (2016). Inhibition of Chikungunya Virus-Induced Cell Death by Salicylate-Derived Bryostatin Analogues Provides Additional Evidence for a PKC-Independent Pathway. J. Nat. Prod..

[129] Pu S.-Y., Wouters R., Schor S., Rozenski J., Barouch-Bentov R., Prugar L.I., O’brien C.M., Brannan J.M., Dye J.M., Herdewijn P. (2018). Optimization of Isothiazolo[4,3-b]pyridine-Based Inhibitors of Cyclin G Associated Kinase (GAK) with Broad-Spectrum Antiviral Activity. J. Med. Chem..

[130] Mounce B.C., Cesaro T., Moratorio G., Hooikaas P.J., Yakovleva A., Werneke S.W., Smith E.C., Poirier E.Z., Simon-Loriere E., Prot M. (2016). Inhibition of Polyamine Biosynthesis Is a Broad-Spectrum Strategy against RNA Viruses. J. Virol..

[131] Cheung Y.Y., Chen K.C., Chen H., Seng E.K., Chu J.J.H. (2014). Antiviral activity of lanatoside C against dengue virus infection. Antivir. Res..

[132] Müller M., Slivinski N., Todd E.J.A.A., Khalid H., Li R., Karwatka M., Merits A., Mankouri J., Tuplin A. (2019). Chikungunya virus requires cellular chloride channels for efficient genome replication. PLoS Negl. Trop. Dis..

[133] Bouma E.M., van de Pol D.P.I., Sanders I.D., Rodenhuis-Zybert I.A., Smit J.M. (2020). Serotonergic Drugs Inhibit Chikungunya Virus Infection at Different Stages of the Cell Entry Pathway. J. Virol..

[134] Mainou B.A., Ashbrook A.W., Smith E.C., Dorset D.C., Denison M.R., Dermody T.S. (2015). Serotonin Receptor Agonist 5-Nonyloxytryptamine Alters the Kinetics of Reovirus Cell Entry. J. Virol..

[135] Ekins S., Madrid P.B. (2020). Tilorone, a broad-spectrum antiviral for emerging viruses. Antimicrob. Agents Chemother..

[136] Gall B., Pryke K., Abraham J., Mizuno N., Botto S., Sali T.M., Broeckel R., Haese N., Nilsen A., Placzek A. (2017). Emerging Alphaviruses Are Sensitive to Cellular States Induced by a Novel Small-Molecule Agonist of the STING Pathway. J. Virol..

[137] Sali T.M., Pryke K.M., Abraham J., Liu A., Archer I., Broeckel R., Staverosky J.A., Smith J.L., Al-Shammari A., Amsler L. (2015). Characterization of a Novel Human-Specific STING Agonist that Elicits Antiviral Activity Against Emerging Alphaviruses. PLoS Pathog..

[138] Pryke K.M., Abraham J., Sali T.M., Gall B.J., Archer I., Liu A., Bambina S., Baird J., Gough M., Chakhtoura M. (2017). A novel agonist of the trif pathway induces a cellular state refractory to replication of Zika, Chikungunya, and dengue viruses. MBio.

[139] Herrero L.J., Foo S.-S., Sheng K.-C., Chen W., Forwood M.R., Bucala R., Mahalingam S. (2015). Pentosan Polysulfate: A Novel Glycosaminoglycan-Like Molecule for Effective Treatment of Alphavirus-Induced Cartilage Destruction and Inflammatory Disease. J. Virol..

[140] Supramaniam A., Liu X., Ferro V., Herrero L.J. (2018). Prophylactic antiheparanase activity by PG545 is antiviral in vitro and protects against Ross River virus disease in mice. Antimicrob. Agents Chemother..

[141] Pou S., Winter R.W., Nilsen A., Kelly J.X., Li Y., Doggett J.S., Riscoe E.W., Wegmann K.W., Hinrichs D.J., Riscoe M.K. (2012). Sontochin as a guide to the development of drugs against chloroquine-resistant malaria. Antimicrob. Agents Chemother..

[142] Inglot A.D. (1969). Comparison of the Antiviral Activity in vitro of some Non-steroidal Anti-inflammatory Drugs. J. Gen. Virol.

[143] Shimizu Y., Yamamoto S., Homma M., Ishida N. (1972). Effect of chloroquine on the growth of animal viruses. Arch. Gesamte Virusforsch..

[144] Helenius A., Marsht M., Whiter J. (1982). Inhibition of Semliki Forest Virus Penetration by Lysosomotropic Weak Bases. J. Gen. Virol.

[145] Cassell S., Edwards J., Brown D.T. (1984). Effects of lysosomotropic weak bases on infection of BHK-21 cells by Sindbis virus. J. Virol..

[146] Freedman A., Steinberg V.L. (1960). Chloroquine in rheumatoid arthritis; a double blindfold trial of treatment for one year. Ann. Rheum. Dis.

[147] Brighton S.W. (1984). Chloroquine phosphate treatment of chronic Chikungunya arthritis An open pilot study. S. Afr. Med. J..

[148] De Lamballerie X., Boisson V., Reynier J.-C., Enault S., Charrel R.N., Flahault A., Roques P., Le Grand R. (2008). On Chikungunya Acute Infection and Chloroquine Treatment. Vector-Borne Zoonotic Dis..

[149] Thiberville S.D., Boisson V., Gaudart J., Simon F., Flahault A., de Lamballerie X. (2013). Chikungunya Fever: A Clinical and Virological Investigation of Outpatients on Reunion Island, South-West Indian Ocean. PLoS Negl. Trop. Dis..

[150] Chopra A., Saluja M., Venugopalan A. (2014). Effectiveness of chloroquine and inflammatory cytokine response in patients with early persistent musculoskeletal pain and arthritis following chikungunya virus infection. Arthritis Rheumatol..

[151] Rodrigo C., Fernando S.D., Rajapakse S., Paul M. (2020). Systematic review Clinical evidence for repurposing chloroquine and hydroxychloroquine as antiviral agents: A systematic review. Clin. Microbiol. Infect..

[152] Roques P., Thiberville S.D., Dupuis-Maguiraga L., Lum F.M., Labadie K., Martinon F., Gras G., Lebon P., Ng L.F.P., de Lamballerie X. (2018). Paradoxical effect of chloroquine treatment in enhancing chikungunya virus infection. Viruses.

[153] Ozden S., Lucas-Hourani M., Ceccaldi P.-E., Basak A., Valentine M., Benjannet S., Hamelin J., Jacob Y., Mamchaoui K., Mouly V. (2008). Inhibition of Chikungunya virus infection in cultured human muscle cells by furin inhibitors: Impairment of the maturation of the E2 surface glycoprotein. J. Biol. Chem..

[154] Faraone I., Labanca F., Ponticelli M., De Tommasi N., Milella L. (2020). Recent Clinical and Preclinical Studies of Hydroxychloroquine on RNA Viruses and Chronic Diseases: A Systematic Review. Molecules.

[155] Padmakumar B., Jayan J.B., Menon R.M.R., Krishnankutty B., Payippallil R., Nisha R.S. (2009). Comparative evaluation of four therapeutic regimes in Chikungunya arthritis: A prospective randomized parallel-group study. Indian J. Rheumatol..

[156] Ravindran V., Alias G. (2017). Efficacy of combination DMARD therapy vs. hydroxychloroquine monotherapy in chronic persistent chikungunya arthritis: A 24-week randomized controlled open label study. Clin. Rheumatol..

[157] Pandya S. (2008). Methotrexate and hydroxychloroquine combination therapy in chronic chikungunya arthritis: A 16 week study. Indian J. Rheumatol..

[158] Bouquillard E., Fianu A., Bangil M., Charlette N., Ribéra A., Michault A., Favier F., Simon F., Flipo R.M. (2018). Rheumatic manifestations associated with Chikungunya virus infection: A study of 307 patients with 32-month follow-up (RHUMATOCHIK study). Jt. Bone Spine.

[159] Sourisseau M., Schilte C., Casartelli N., Trouillet C., Guivel-Benhassine F., Rudnicka D., Sol-Foulon N., Le Roux K., Prevost M.-C., Fsihi H. (2007). Characterization of Reemerging Chikungunya Virus. PLoS Pathog..

[160] Irurzun A., Nieva J.L., Carrasco L. (1997). Entry of Semliki forest virus into cells: Effects of concanamycin A and nigericin on viral membrane fusion and infection. Virology.

[161] Jurgeit A., Mcdowell R., Moese S., Meldrum E., Schwendener R. (2012). Niclosamide Is a Proton Carrier and Targets Acidic Endosomes with Broad Antiviral Effects. PLoS Pathog..

[162] Koivusalo M., Welch C., Hayashi H., Scott C.C., Kim M., Alexander T., Touret N., Hahn K.M., Grinstein S. (2010). Amiloride inhibits macropinocytosis by lowering submembranous pH and preventing Rac1 and Cdc42 signaling. J. Cell Biol..

[163] Shu Q., Nair V. (2008). Inosine monophosphate dehydrogenase (IMPDH) as a target in drug discovery. Med. Res. Rev..

[164] Turner T.L., Kopp B.T., Paul G., Landgrave L.C., Hayes D., Thompson R. (2014). Respiratory syncytial virus: Current and emerging treatment options. Clin. Outcomes Res..

[165] Paeshuyse J., Dallmeier K., Neyts J. (2011). Ribavirin for the treatment of chronic hepatitis C virus infection: A review of the proposed mechanisms of action. Curr. Opin. Virol..

[166] Gallegos K.M., Drusano G.L., D′Argenio D.Z., Brown A.N. (2016). Chikungunya Virus: In Vitro Response to Combination Therapy With Ribavirin and Interferon Alfa 2a. J. Infect. Dis..

[167] Rothan H.A., Bahrani H., Abdulrahman A.Y., Mohamed Z., Teoh T.C., Othman S., Rashid N.N., Rahman N.A., Yusof R. (2016). Mefenamic acid in combination with ribavirin shows significant effects in reducing chikungunya virus infection in vitro and in vivo. Antivir. Res..

[168] Ravichandran R., Manian M. (2008). Ribavirin therapy for Chikungunya arthritis. J. Infect. Dev. Ctries..

[169] Rashad A.A., Neyts J., Leyssen P., Keller P.A. (2018). A reassessment of mycophenolic acid as a lead compound for the development of inhibitors of chikungunya virus replication. Tetrahedron.

[170] Khan M., Dhanwani R., Patro I.K., Rao P.V.L., Parida M.M. (2010). Cellular IMPDH enzyme activity is a potential target for the inhibition of Chikungunya virus replication and virus induced apoptosis in cultured mammalian cells. Antivir. Res..

[171] Rada B., Dragúň M. (1977). Antiviral Action and Selectivity of 6-Azauridine. Ann. N. Y. Acad. Sci..

[172] Deneau D.G., Farber E.M. (1975). The treatment of psoriasis with azaribine. Dermatologica.

[173] Cruthcher W.A., Moschella S.L. (1975). Double-blind controlled crossover high-dose study of Azaribine in psoriasis. Br. J. Dermatol..

[174] Raveh A., Delekta P.C., Dobry C.J., Peng W., Schultz P.J., Blakely P.K., Tai A.W., Matainaho T., Irani D.N., Sherman D.H. (2013). Discovery of Potent Broad Spectrum Antivirals Derived from Marine Actinobacteria. PLoS ONE.

[175] Ashton T.M., Fokas E., Kunz-Schughart L.A., Folkes L.K., Anbalagan S., Huether M., Kelly C.J., Pirovano G., Buffa F.M., Hammond E.M. (2016). The anti-malarial atovaquone increases radiosensitivity by alleviating tumour hypoxia. Nat. Commun..

[176] Keller T.L., Zocco D., Sundrud M.S., Hendrick M., Edenius M., Yum J., Kim Y.-J., Lee H.-K., Cortese J.F., Wirth D.F. (2012). Halofuginone and other febrifugine derivatives inhibit prolyl-tRNA synthetase. Nat. Chem. Biol..

[177] Fresno M., Jiménez A., Vázquez D. (1977). Inhibition of Translation in Eukaryotic Systems by Harringtonine. Eur. J. Biochem..

[178] McKendrick L., Morley S.J., Pain V.M., Jagus R., Joshi B. (2001). Phosphorylation of eukaryotic initiation factor 4E (eIF4E) at Ser209 is not required for protein synthesis in vitro and in vivo. Eur. J. Biochem..

[179] Henss L., Scholz T., Grünweller A., Schnierle B., Henss L., Scholz T., Grünweller A., Schnierle B.S. (2018). Silvestrol Inhibits Chikungunya Virus Replication. Viruses.

[180] Blum L., Geisslinger G., Parnham M.J., Grünweller A., Schiffmann S. (2020). Natural antiviral compound silvestrol modulates human monocyte-derived macrophages and dendritic cells. J. Cell. Mol. Med..

[181] Luo H. (2016). Interplay between the virus and the ubiquitin-proteasome system: Molecular mechanism of viral pathogenesis. Curr. Opin. Virol..

[182] Geller R., Taguwa S., Frydman J. (2012). Broad action of Hsp90 as a host chaperone required for viral replication. Biochim. Biophys. Acta Mol. Cell Res..

[183] Das I., Basantray I., Mamidi P., Nayak T.K., Pratheek B.M., Chattopadhyay S., Chattopadhyay S. (2014). Heat Shock Protein 90 Positively Regulates Chikungunya Virus Replication by Stabilizing Viral Non-Structural Protein nsP2 during Infection. PLoS ONE.

[184] Izumida M., Hayashi H., Tanaka A., Kubo Y. (2020). Cathepsin B Protease Facilitates Chikungunya Virus Envelope Protein-Mediated Infection Via Endocytosis or Macropinocytosis. Viruses.

[185] Klimstra W.B., Heidner H.W., Johnston R.E., Klimstra B., Ryman K.D., Johnston R.E., Virol J. (1999). The Furin Protease Cleavage Recognition Sequence of Sindbis Virus PE2 Can Mediate Virion Attachment to Cell Surface Heparan Sulfate. J. Virol..

[186] Heidner H.W., Knott T.A., Johnston R.E., Heidner H.W., McKnight K.L., Davis N.L., Johnston R.E., Virol J. (1996). Differential Processing of Sindbis Virus Glycoprotein PE2 in Cultured Vertebrate and Arthropod Cells. J. Virol..

[187] Zhang X., Fugère M., Day R., Kielian M. (2003). Furin Processing and Proteolytic Activation of Semliki Forest Virus. J. Virol..

[188] Hardes K., Ivanova T., Thaa B., McInerney G.M., Klokk T.I., Sandvig K., Künzel S., Lindberg I., Steinmetzer T. (2017). Elongated and Shortened Peptidomimetic Inhibitors of the Proprotein Convertase Furin. ChemMedChem.

[189] Wolfe M.S., Borchardt R.T. (1991). S-Adenosyl-L-homocysteine Hydrolase as a Target for Antiviral Chemotherapy. J. Med. Chem..

[190] Keating J.A., Striker R. (2012). Phosphorylation events during viral infections provide potential therapeutic targets. Rev. Med. Virol..

[191] Wan J.J., Brown R.S., Kielian M. (2020). Berberine chloride is an alphavirus inhibitor that targets nucleocapsid assembly. MBio.

[192] Bourjot M., Delang L., Nguyen V.H., Neyts J., Oise Gueitte F., Leyssen P., Litaudon M. (2012). Prostratin and 12-O-Tetradecanoylphorbol 13-Acetate Are Potent and Selective Inhibitors of Chikungunya Virus Replication. J. Nat. Prod..

[193] Abdelnabi R., Staveness D., Near K.E., Wender P.A., Delang L., Neyts J., Leyssen P. (2016). Comparative analysis of the anti-chikungunya virus activity of novel bryostatin analogs confirms the existence of a PKC-independent mechanism. Biochem. Pharmacol..

[194] Mounce B.C., Poirier E.Z., Passoni G., Simon-Loriere E., Cesaro T., Prot M., Stapleford K.A., Moratorio G., Sakuntabhai A., Levraud J.-P. (2016). Interferon-Induced Spermidine-Spermine Acetyltransferase and Polyamine Depletion Restrict Zika and Chikungunya Viruses. Cell Host Microbe.

[195] Mounce B.C., Cesaro T., Vlajnić L., Vidiņa A., Vallet T., Weger-Lucarelli J., Passoni G., Stapleford K.A., Levraud J.-P., Vignuzzi M. (2017). Chikungunya Virus Overcomes Polyamine Depletion by Mutation of nsP1 and the Opal Stop Codon To Confer Enhanced Replication and Fitness. J. Virol..

[196] Hover S., Foster B., Barr J.N., Mankouri J. (2017). Viral dependence on cellular ion channels—An emerging antiviral target?. J. Gen. Virol..

[197] Cook L.E., Locke M.C., Young A.R., Monte K., Hedberg M.L., Shimak R.M., Sheehan K.C.F., Veis D.J., Diamond M.S., Lenschow D.J. (2019). Distinct Roles of Interferon Alpha and Beta in Controlling Chikungunya Virus Replication and Modulating Neutrophil-Mediated Inflammation. J. Virol..

[198] Krueger R.F., Mayer G.D. (1970). Tilorone hydrochloride: An orally active antiviral agent. Science.

[199] Li Y.G., Siripanyaphinyo U., Tumkosit U., Noranate N., A-Nuegoonpipat A., Pan Y., Kameoka M., Kurosu T., Ikuta K., Takeda N. (2012). Poly (I:C), an agonist of toll-like receptor-3, inhibits replication of the Chikungunya virus in BEAS-2B cells. Virol. J..

[200] Priya R., Patro I.K., Parida M.M. (2014). TLR3 mediated innate immune response in mice brain following infection with Chikungunya virus. Virus Res..

[201] Ichinohe T., Watanabe I., Ito S., Fujii H., Moriyama M., Tamura S., Takahashi H., Sawa H., Chiba J., Kurata T. (2005). Synthetic Double-Stranded RNA Poly(I:C) Combined with Mucosal Vaccine Protects against Influenza Virus Infection. J. Virol..

[202] Beljanski V., Chiang C., Kirchenbaum G.A., Olagnier D., Bloom C.E., Wong T., Haddad E.K., Trautmann L., Ross T.M., Hiscott J. (2015). Enhanced Influenza Virus-Like Particle Vaccination with a Structurally Optimized RIG-I Agonist as Adjuvant. J. Virol..

[203] Goulet M.-L., Olagnier D., Xu Z., Paz S., Belgnaoui S.M., Lafferty E.I., Janelle V., Arguello M., Paquet M., Ghneim K. (2013). Systems Analysis of a RIG-I Agonist Inducing Broad Spectrum Inhibition of Virus Infectivity. PLoS Pathog..

[204] Olagnier D., Scholte F.E.M., Chiang C., Albulescu I.C., Nichols C., He Z., Lin R., Snijder E.J., van Hemert M.J., Hiscott J. (2014). Inhibition of Dengue and Chikungunya Virus Infections by RIG-I-Mediated Type I Interferon-Independent Stimulation of the Innate Antiviral Response. J. Virol..

[205] Chiang C., Beljanski V., Yin K., Olagnier D., Ben Yebdri F., Steel C., Goulet M.-L., DeFilippis V.R., Streblow D.N., Haddad E.K. (2015). Sequence-Specific Modifications Enhance the Broad-Spectrum Antiviral Response Activated by RIG-I Agonists. J. Virol..

[206] Teng T.S., Foo S.S., Simamarta D., Lum F.M., Teo T.H., Lulla A., Yeo N.K.W., Koh E.G.L., Chow A., Leo Y.S. (2012). Viperin restricts chikungunya virus replication and pathology. J. Clin. Investig..

[207] Carissimo G., Teo T.H., Chan Y.H., Lee C.Y.P., Lee B., Torres-Ruesta A., Tan J.J.L., Chua T.K., Fong S.W., Lum F.M. (2019). Viperin controls chikungunya virus-specific pathogenic T cell IFNγ Th1 stimulation in mice. Life Sci. Alliance.

[208] Hammond E., Haynes N.M., Cullinane C., Brennan T.V., Bampton D., Handley P., Karoli T., Lanksheer F., Lin L., Yang Y. (2018). Immunomodulatory activities of pixatimod: Emerging nonclinical and clinical data, and its potential utility in combination with PD-1 inhibitors. J. Immunother. Cancer.

[209] Dredge K., Brennan T.V., Hammond E., Lickliter J.D., Lin L., Bampton D., Handley P., Lankesheer F., Morrish G., Yang Y. (2018). A Phase i study of the novel immunomodulatory agent PG545 (pixatimod) in subjects with advanced solid tumours. Br. J. Cancer.

[210] Krishnan R., Duiker M., Rudd P.A., Skerrett D., D Pollard J.G., Siddel C., Rifat R., K Ng J.H., Georgius P., Hererro L.J. (2021). Pentosan polysulfate sodium for Ross River virus-induced arthralgia: A phase 2a, randomized, double-blind, placebo-controlled study. BMC Musculoskelet. Disord..

[211] Marra R.K.F., Kümmerle A.E., Guedes G.P., de Barros S.C., Gomes R.S.P., Cirne-Santos C.C., Paixão I.C.N.P., Neves A.P. (2020). Quinolone-N-acylhydrazone hybrids as potent Zika and Chikungunya virus inhibitors. Bioorganic Med. Chem. Lett..

[212] Ching K.-C., Kam Y.-W., Merits A., Ng L.F.P., Chai C.L.L. (2015). Trisubstituted Thieno[3,2-b]pyrrole 5-Carboxamides as Potent Inhibitors of Alphaviruses. J. Med. Chem..

[213] Ching K.-C., Ngoc Quy Tran T., Naqiah Amrun S., Kam Y.-W., P Ng L.F., L Chai C.L. (2017). Structural Optimizations of Thieno[3,2-b]pyrrole Derivatives for the Development of Metabolically Stable Inhibitors of Chikungunya Virus. J. Med. Chem..

[214] Fares M., McCosker P.M., Alsherbiny M.A., Willis A.C., Clark T., Neyts J., Jochmans D., Keller P.A. (2020). Regioselective convergent synthesis of 2-arylidene thiazolo[3,2- a] pyrimidines as potential anti-chikungunya agents. RSC Adv..

[215] Hwu J.R., Kapoor M., Tsay S.-C., Lin C.-C., Hwang K.C., Horng J.-C., Chen I.-C., Shieh F.-K., Leyssen P., Neyts J. (2015). Benzouracil–coumarin–arene conjugates as inhibiting agents for chikungunya virus. Antivir. Res..

